# Efficacy and safety of neoadjuvant PD-1 inhibitors or PD-L1 inhibitors combined with chemoradiotherapy for locally advanced rectal cancer: a systematic review and meta-analysis

**DOI:** 10.3389/fphar.2025.1570467

**Published:** 2025-05-16

**Authors:** Jiaojiao Yu, Zhijing Wang, Mingxu Li, Hua Zhu, Xiaoxia Tang, Ke Luan, Yinhuan Zhi, Shan Yin, Yuanqi Su, Jingyan Long, Qubo He, Jieru Quan, Chenchen Li

**Affiliations:** ^1^ Department of Medical Oncology, The Sixth Affiliated Hospital, Sun Yat-sen University, Guangzhou, Guangdong, China; ^2^ The First Affiliated Hospital of Guangxi University of Science and Technology, Guangxi University of Science and Technology, Liuzhou, Guangxi, China; ^3^ Department of Urology, Pu’er People’s Hospital, Pu’er, Yunnan, China; ^4^ School of Economics and Management, Guangxi University of Science and Technology, Liuzhou, Guangxi, China; ^5^ Guangdong Provincial Key Laboratory of Colorectal and Pelvic Floor Diseases, Guangzhou, Guangdong, China; ^6^ Biomedical Innovation Center, The Sixth Affiliated Hospital, Sun Yat-sen University, Guangzhou, Guangdong, China; ^7^ State Key Laboratory of Oncology in South China, Guangzhou, Guangdong, China

**Keywords:** PD-1 inhibitor, programmed cell death protein 1 inhibitor, programmed death-ligand 1 inhibitor, rectal cancer, neoadjuvant, chemoradiotherapy, meta‐analysis

## Abstract

**Background:**

The objective of this meta-analysis was to assess the effectiveness and safety of neoadjuvant PD-1/L1 inhibitors plus chemoradiotherapy(CRT) for locally advanced rectal cancer (LARC).

**Materials and Methods:**

Databases including PubMed, Embase, Cochrane Library and Web of Science were examined for pertinent studies. Meta-analyses were conducted on pathological complete response (pCR), clinical complete response (cCR), major pathologic response (MPR), sphincter-sparing surgery (SSS), R0 resection, surgery rate, Grade≥3 adverse events (AEs), and 3-year disease-free survival (DFS).

**Results:**

The combined percentages of pCR, cCR, MPR, SSS, R0 resection rate, surgery rate, and 3-year DFS were 30.8%, 20.8%, 57.6%, 70.3%, 75.8%, 83.5%, and 76%, respectively. Grade ≥3 AEs manifested in 33.9% of cases. In subgroup analysis, mismatch repair-deficient (dMMR) or microsatellite instability-high (MSI-H) showed 50.2% pCR and 64.7% MPR. Long-course radiotherapy (LCRT) and short-course radiotherapy (SCRT) had 39.1% and 27.1% pCR rates. The contemporaneous and sequential immuno-chemoradiotherapy subgroups had 30.8% and 30.1% pCR rates. These rates matched the 33.1% and 30% pCR rates for the PD-L1 and PD-1 inhibitor subgroups. The PD-L1 and PD-1 inhibitor categories had 20.6% and 38.8% rate of Grade ≥3AEs.

**Conclusion:**

Neoadjuvant PD-1/PD-L1 inhibitors plus CRT have demonstrated favourable response rates and tolerable toxicity profiles for LARC.

**Systematic Review Registration:**

PROSPERO (CRD42024569289) https://www.crd.york.ac.uk/prospero/display_record.php?ID=CRD42024569289.

## 1 Introduction

Rectal cancer is a prevalent malignant tumour observed in the gastrointestinal tract (Wang YF. et al., 2022). Rectal cancer is the third most prevalent malignant tumour in males and the second in women worldwide, according to 2020 epidemiological figures from the International Agency for Research on Cancer (IARC). America, Australia, and New Zealand had the most cases, with 392,000 in China in 2018 (Wang YF. et al., 2022). The National Cancer Institute (USA) has provided figures indicating that the 5-year overall survival (OS) rates for rectal malignancies rate at 67% (Zhu et al., 2023). Rectal cancer is often diagnosed at late stages, worsening the problem.

The conventional neoadjuvant treatment methods consist of extended chemoradiotherapy (50 Gy/25Fx) performed in combination with 5-FU or Capecitabine, as well as short-course radiation (25 Gy/5Fx) (WA, 2019). According to the National Comprehensive Cancer Network (NCCN) recommendations, the preferred standard treatment for reducing recurrence rate is neoadjuvant CRT followed by total mesorectal excision (TME) (Saraf et al., 2022; Willett, 2018). This treatment intervention has the potential to greatly decrease the 5-year local recurrence rate to 5%–10% in patients with LARC (Chen et al., 2024). Nevertheless, the distant metastatic rate does not demonstrate any substantial reduction and stays as high as 35%, resulting in cancer progression and unfavourable prognosis. Only 11%–15% of patients successfully achieve pCR after neoadjuvant chemotherapy (Breugom et al., 2015; Bosset et al., 2005; Gérard et al., 2006). LARC patients may experience improved disease control rates, reduced comorbidities, enhanced quality of life, organ preservation, and better oncological prognosis (Yang et al., 2024c). Therefore, there is an urgent pressing need for more efficient neoadjuvant treatments against LARC (Yang et al., 2024c).

The research on immune checkpoint inhibitors (ICIs) in different types of tumours has been rapidly expanding in recent years. The findings have consistently demonstrated that ICIs have a valuable therapeutic impact (Wang YF. et al., 2022). Immunotherapy is purported to greatly enhance the outlook of patients with colorectal cancer, and ICIs have also been suggested as a primary choice for managing advanced rectal cancer (Garcia-Aguilar et al., 2015; Conroy et al., 2021; Cercek et al., 2018; André et al., 2020a; Lichthardt et al., 2017; Sclafani et al., 2017; Zhang et al., 2019a; Hasegawa et al., 2017). Previous research indicates that ICIs have positive outcomes in the neoadjuvant therapy of resectable rectal cancer. Additionally, ICI medicines have significant promise in the holistic treatment decision-making for locally progressed and early rectal cancer (Wang YF. et al., 2022). In 2022, sindilizumab, a novel PD-1 inhibitor medication, was officially incorporated into the CSCO guidelines for the clinical use of ICIs. This milestone marked the inclusion of all first-line treatments for five major tumours in the CSCO guidelines (Toritani et al., 2020; Eisterer et al., 2017). In recent years, numerous clinical trials have explored the efficacy and safety of PD-1 inhibitors in the treatment of advanced colorectal cancer, revealing favorable responses in a subset of patients, which has stimulated our interest in this therapeutic approach. A recent study by PICC, published in Lancet Gastroenterol Hepatol in 2021, proposed that the pCR rate for initially resectable dMMR/MSI-H LACRC treated with toripalimab alone might approach 65% (Hu et al., 2022). In contrast, the KEYNOTE-177 trial revealed that 29.4% of patients with dMMR/MSI-H, metastatic, colorectal cancer who received first-line pembrolizumab treatment and 12.3% of those treated with chemotherapy, saw disease progression as their ideal response (Zhu et al., 2023). Therefore, neoadjuvant immunotherapy may potentially increase the risk of disease progression in comparison to conventional chemotherapy (Hu et al., 2022). The discrepancies between these two trials might be caused by variations in patient populations, dMMR/MSI-H status or regimens. Notably, single trial is limited by a small sample size, a brief postoperative follow-up time, and the use of a surrogate endpoint. These limitations significantly impact the generalizability and reliability of research findings, including reduced statistical power, heightened vulnerability to random variations, obscured long-term outcomes, diminished ability to detect rare events or complications, and compromised accuracy in treatment effect estimation.

As meta-analyses allow for larger effective sample sizes, they can mitigate the limitations of individual small studies, enabling more robust detection of treatment effects, identification of rare adverse events, and exploration of clinically relevant subgroup differences that may otherwise remain undetected. Therefore, this meta-analysis was designed to increase statistical power by pooling data from multiple trials, in order to provide more reliable evidence about the efficacy and safety of neoadjuvant PD-1 inhibitors or PD-L1 inhibitors combined with chemoradiotherapy for locally advanced rectal cancer.

## 2 Material and methods

### 2.1 Search strategy

The 2020 PRISMA guidelines were used to design this meta-analysis. This work is registered at PROSPERO under CRD42024569289. A complete search of PubMed, Embase, Web of Science, and the Cochrane Library was done to find literature published until 1 September 2024. The search approach was (“PD-1 inhibitor” OR “PD-L1 inhibitor” OR “immune checkpoint inhibitors” OR “immunotherapy”) AND “neoadjuvant” AND “rectal cancer” AND “trial”. We also performed a thorough manual examination of the bibliographies of the identified articles, in order to uncover any new studies that fit the criteria for inclusion. Supplementary Material S1 offered a thorough summary of the search record.

### 2.2 Inclusion and exclusion criteria

Inclusion criteria: (1) Patients diagnosed as LARC; (2) Patients of at least one group received neoadjuvant PD-(L)1 inhibitors plus chemoradiotherapy; (3) At least one of the following outcomes was reported: pCR, cCR, MPR, SSS, R0 resection rate, surgery rate, 3-year DFS and Grade≥3 AEs; (4) Types of studies: randomised controlled trials (RCTs), single-arm trials, prospective studies.

Exclusion criteria: (1) Articles of other types, such as case reports, publications, letters, reviews, meta-analyses, editorials, animal studies, and protocols; (2) Duplicate records; (3) Not relevant; (4) Duplicate cohort; (5) Failed to obtain full-text; (6) Failed to extract data; (7) non-English articles.

### 2.3 Selection of studies

Selection of studies, including elimination of duplicates, was undertaken using EndNote (Version 20; Clarivate Analytics). An initial search was undertaken by two reviewers who independently deleted duplicate entries, assessed the titles and abstracts for relevance, and classified each study as either included or excluded. The settlement was arrived at through the attainment of consensus. A third author of the review would take on the role of an arbitrator if lacking a consensus.

### 2.4 Data extraction

After reviewing the title and abstract, two independent reviewers proceeded to study the complete content. Consultation with a third investigator settled the discrepancy. The retrieved data comprised the first author’s name, year, trial ID, study design, sample size, intervention, male ratio, age, stage, regimen, pCR, cCR, MPR, SSS, R0 resection, Grade≥3 AEs and 3-year DFS. Regarding studies with incomplete or missing data for baseline characteristics and outcomes, attempts were made to contact the authors to obtain relative information. Studies would be excluded if none of the listed outcomes was obtained after attempts.

### 2.5 Risk of bias assessment

Dual independent reviewers evaluated the risk of bias using the modified Jadad scale for randomised controlled trials and the methodological index for non-randomized studies (MINORS) for single-arm and prospective studies.

### 2.6 Statistical analysis

A duplicate removal process was performed on the included studies using EndNote (Version 20; Clarivate Analytics). All statistical analyses were conducted using Stata 18.0. The analysis using the “metaprop” utility package. The continuous variables were compared using the weighted mean difference (WMD) method, with a 95% statistical confidence interval (CI). Comparison of binary variables was conducted using relative ratio (RR) with a 95% confidence interval (CI). The mean and standard deviation were derived using the medians and interquartile ranges of continuous data. The Cochrane ‘Sq test and the I^2^ index were used to compute statistical heterogeneity among the included studies. The choice between fixed - effect and random - effect models was based on the I^2^ value and chi-square test P value. When heterogeneity was high (I^2^ >50%), the random - effect model was used. When heterogeneity was low (I^2^ ≤ 50%), the fixed - effect model was applicable. Any p value less than 0.05 was deemed statistically significant. Publication bias was assessed using funnel plot. Sensitivity analysis systematically assessed the impact of heterogeneity on pooled effect estimates and overall conclusions. Meta-regression analysis evaluated the significance of subgroup effects.

## 3 Results

### 3.1 Search results

**FIGURE 1:**
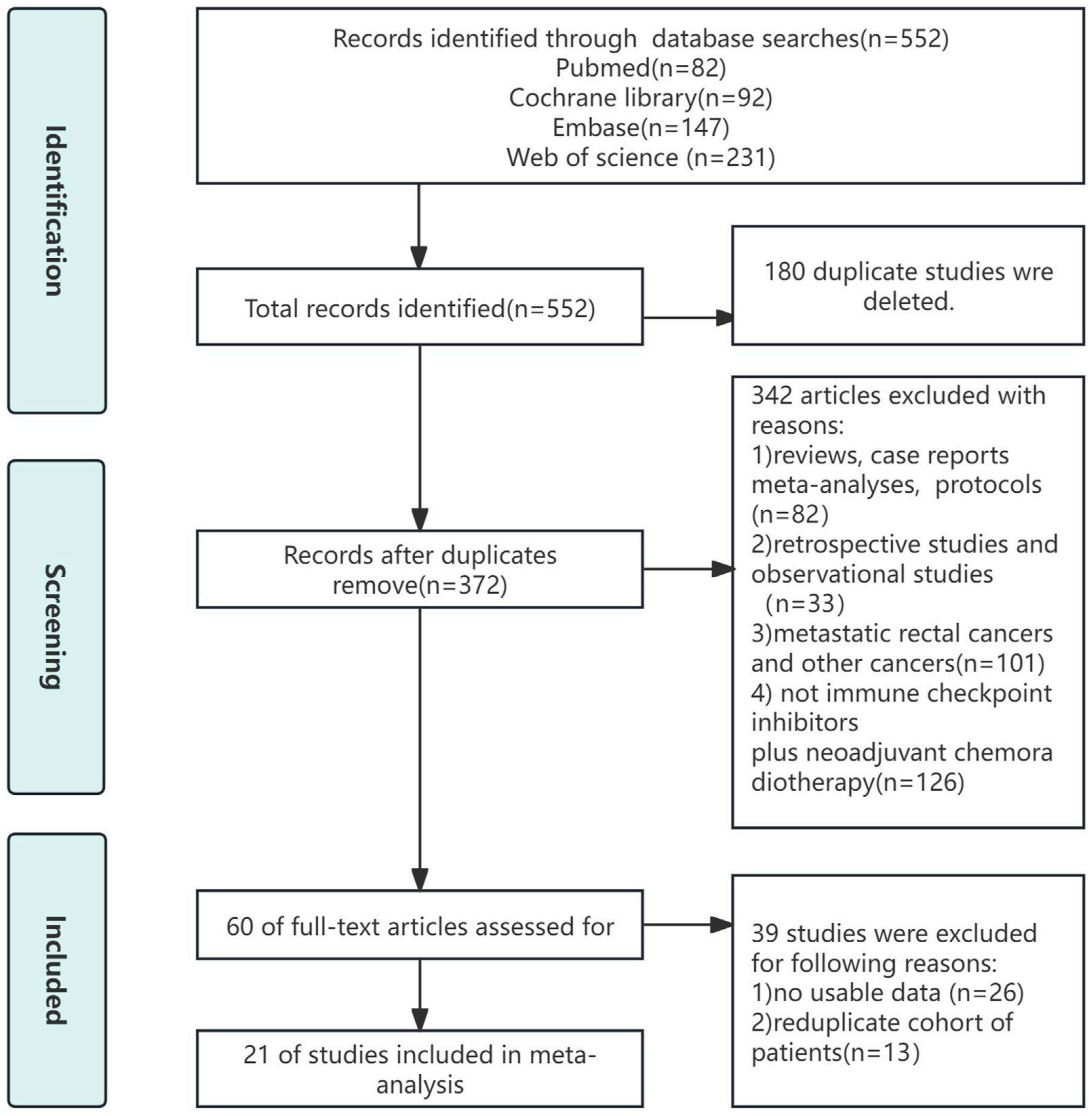
Flow chart of literature search strategies.

The procedure for selecting and including literature was illustrated in Figure 1. Our preliminary study yielded a grand total of 284 publications. After removing duplicate studies, the remaining number of cases was mere 185. Upon thorough examination of the complete text, 42 papers of different types, 103 irrelevant articles, and 10 retrospective studies were eliminated. Ultimately, this meta-analysis included 21 studies.

### 3.2 Patient characteristics and quality assessment

This meta-analysis included 21 studies, most of which were phase Ⅱ/Ⅲ single-arm studies. Patients at least one group received neoadjuvant PD-(L)1 inhibitors combined with chemoradiotherapy (one pembrolizumab + CRT (Rahma et al., 2021), three camrelizumab + CRT (Lin et al., 2021; Li et al., 2024; Lin et al., 2024), three durvalumab + CRT (Castillon et al., 2023; George et al., 2022; Grassi et al., 2023), four sintilimab + CRT (Zhou et al., 2022; Jiao et al., 2023; Xiao et al., 2024; Liu et al., 2024), one atezolizumab + CRT (Carrasco et al., 2023), two avelumab + CRT (Shamseddine et al., 2022; Salvatore et al., 2021), two tislelizumab + CRT (Yang et al., 2024a; Yang Y. et al., 2024), two nivolumab + CRT (Bando et al., 2022a; Brenner et al., 2024), one toripalimab + CRT (Xia et al., 2024) and two Envafolimab + CRT (Dai et al., 2023; Wang et al., 2024)). A modified Jadad scale was used to assess the quality of RCTs, and all five RCTs were classified as high-quality papers. Other articles were scored using MINORS, with 15 points for 1 article, 14 points for six articles, 13 points for six articles, and 12 points for 3 articles. The information concerning patient characteristics and quality assessment is provided in Table 1. Detailed information regarding the risk of bias assessment for each study is available in Supplementary Material S2.

**TABLE 1:** Characteristics of included studies and patients.

Author	Country	Registration ID	Study design	Stage	Intervention	No. Of patients	Age (median years)	Gender (male %)	Quality
Rahma et al. (2021)	America	NCT02921256	RCT	T3-4N0-3M0	LCRT(50.4Gy)+FOLFOX + pembrolizumab→TME	90	55.7	68.1%	5
Castillon et al. (2023)	America	EudraCT 2018–004835-56	single-arm	Ⅱ/Ⅲ	FOLFOX→CRT + capecitabine + durvalumab→TME	61	61.3	70%	12
Zhou et al. (2022)	China	NCT05215379	single-arm	cT1-3aN0-1M0	LCRT(50Gy)+sintilimab→capecitabine or CAPOX + sintilimab→TME	23	55	61%	14
Lin et al. (2021)	China	NCT04231552	single-arm	cT1-4N + M0	SCRT(25Gy)→CAPOX + Camrelizumab→TME	30	57	56.7%	14
Carrasco et al. (2023)	Belgium	NCT03127007	single-arm	Ⅱ/Ⅲ	LCRT(45–50Gy)+atezolizumab+5FU→TME	39	63	56%	13
Li et al. (2024)	China	NCT04340401	single-arm	NR	CAPOX + camrelizumab + LCRT(50.6Gy)+capecitabine→TME	25	58	76%	13
George et al. (2022)	America	NCT03102047	single-arm	Ⅱ/Ⅲ	LCRT→durvalumab + capecitabine →TME	45	NR	NR	12
Grassi et al. (2023)	Italy	NCT04083365	single-arm	cT2-4N + M0	LCRT(50.4Gy)→durvalumab + capecitabine→TME	60	64	49.1%	14
Shamseddine et al. (2022)	Lebanon	NCT03503630	single-arm	NR	SCRT(25Gy)→mFOLFOX-6+avelumab→TME	40	58.5	65%	14
Bando et al. (2022a)	Japan	NCT02948348	single-arm	cT3–4N0–2 M0	LCRT(50.4Gy)→Nivolumab + capecitabine →TME	44	61	67%	15
Salvatore et al. (2021)	Italy	NCT03854799	single-arm	cT3-4N + M0	LCRT(50.4Gy)+capecitabine + avelumab→TME	101	63	61.4%	13
Lin et al. (2024)	China	NCT04928807	RCT	cT3-4N + M0	SCRT→amrelizumab + CAPOX→TME	113	NR	NR	4
Yang et al. (2024a)	china	NCT04911517	single-arm	cT2-4N0-2M0	LCRT(50Gy)+capecitabine + tislelizumab→TME	38	60.5	53.8%	14
Jiao et al. (2023)	China	NCT05307198	single-arm	NR	Capox→CAPOX + Sintilimab + CRT→TME	20	NR	NR	12
Dai et al. (2023)	China	NCT05216653	single-arm	NR	SCRT→Envafolimab + CAPEOX→TME	21	67	NR	13
Xia et al. (2024)	China	NCT04518280	RCT	cT3-4N + M0	armA:SCRT(25Gy)→CAPOX + toripalimab→TME	62	55	67.7%	5
					armB:CAPOX + toripalimab→SCRT(25Gy)→TME	59	56	67.8%	
Brenner et al. (2024)	Israel	NCT03921684	single-arm	T3-4N + M0, cT + N1M0	LCRT(50.4Gy)→mFOLFOX6+nivolumab→TME	29	53	72%	13
Yang et al. (2024b)	China	NCT05245474	RCT	NR	armA:tislelizumab + capecitabine + CRT→TME	62	NR	NR	5
					armB:LCRT + Tislelizumab + capecitabine→TME	62	NR	NR	
Xiao et al. (2024)	China	NCT04304209	RCT	cT2-4N0-2M0	LCRT + sintilimab + capecitabine→TME	67	56	64.2%	5
Liu et al. (2024)	China	NCT04906044	single-arm	NR	LCRT(50Gy)+capecitabine→CAPEOX + sintilimab→TME	37	59	67.6%	13
Wang et al. (2024)	China	NR	single-arm	cT2N + M0, cT3/T4aN + M0	SCRT(25Gy)→CAPEOX + Envafolimab→TME	32	NR	71.9%	14

RCT: randomised controlled trials; NR: not reported; LCRT: long-course radiotherapy; TME: total mesorectal excision; (m)FOLFOX: 5-fluorouracil, leucovorin, and oxaliplatin; 5FU: 5-Fluorouracil; CRT: chemoradiotherapy; CAPOX: oxaliplatin, capecitabine; SCRT: short-course radiotherapy; CAPEOX: capecitabine, Oxaliplatin.

### 3.3 pCR

**FIGURE 2:**
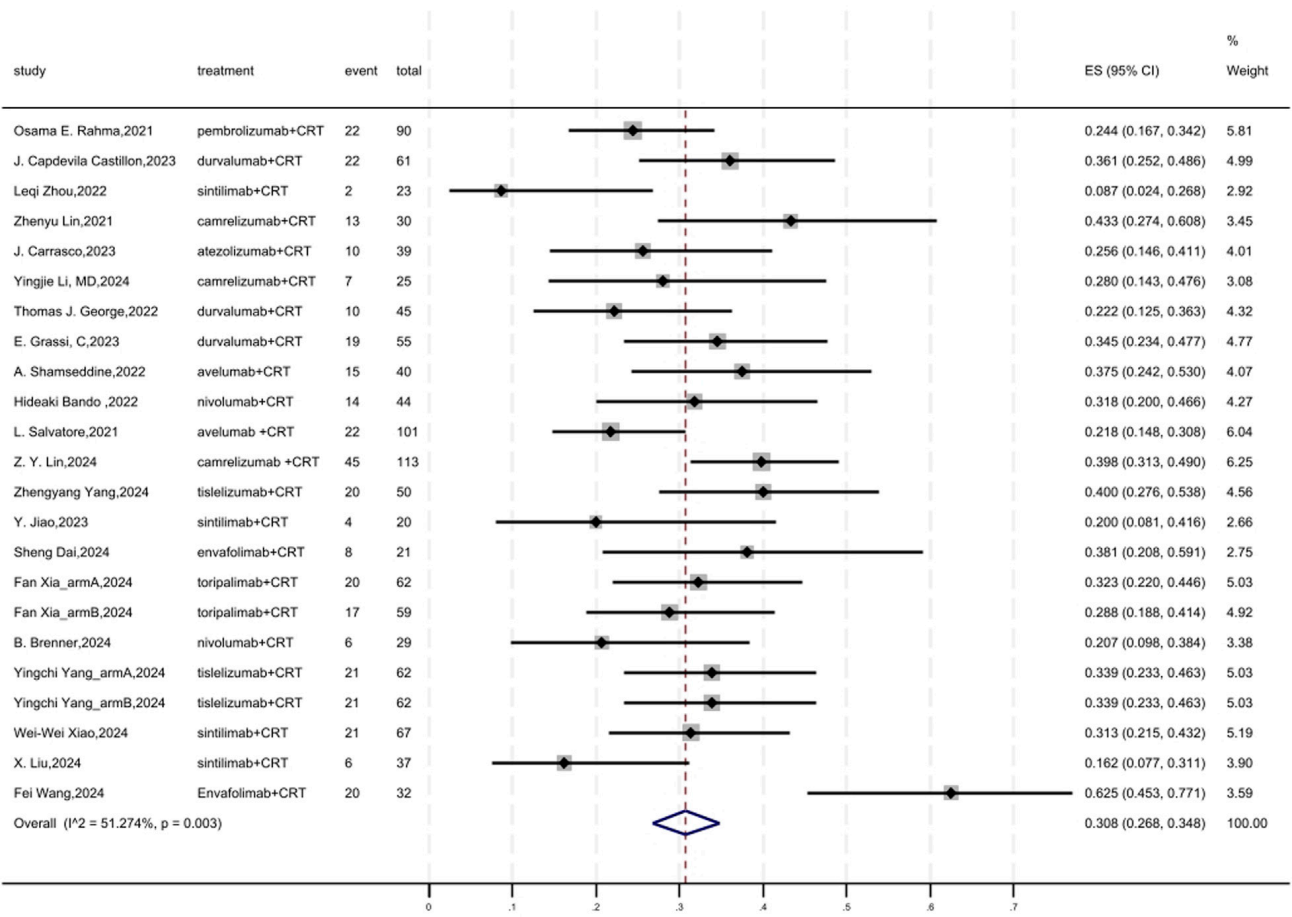
Forest plot of the meta-analysis for pCR.

Figure 2 displayed a forest plot of the meta-analysis focused on pCR. The overall pooled pCR of neoadjuvant PD-(L)1 inhibitors combined with CRT was 0.308(95%CI = 0.268–0.348, I^2^ = 51.274%, P = 0.003). Results of the meta-analysis were shown in Table 2. Sensitivity analysis confirmed the stability of our findings (Supplementary Figure S1). Funnel plot assessment revealed no significant publication bias regarding pCR (Supplementary Figure S2).

**TABLE 2:** Results of the meta-analysis.

0utcomes	No. of studies	Heterogeneity		Overall effect size	95% CI of overall effect
		I^2^ (%)	p-value		
pCR	23	51.274	0.003	0.308	0.268, 0.348
cCR	13	87.377	0.000	0.208	0.124, 0.307
MPR	15	78.353	0.000	0.576	0.498, 0.653
surgery rate	19	90.674	0.000	0.835	0.745, 0.909
R0 resection rate	9	85.150	0.000	0.758	0.651, 0.852
SSS	10	89.966	0.000	0.703	0.569, 0.821
Grade ≥3 AEs	20	94.978	0.000	0.339	0.211, 0.480
3-year DFS	3	70.817	0.032	0.760	0.616, 0.880

pCR: pathological complete response; cCR:clinical complete response; MPR:major pathologic response; SSS: sphincter-sparing surgery; AEs: adverse events; DFS: disease-free survival.

### 3.4 cCR

**FIGURE 3:**
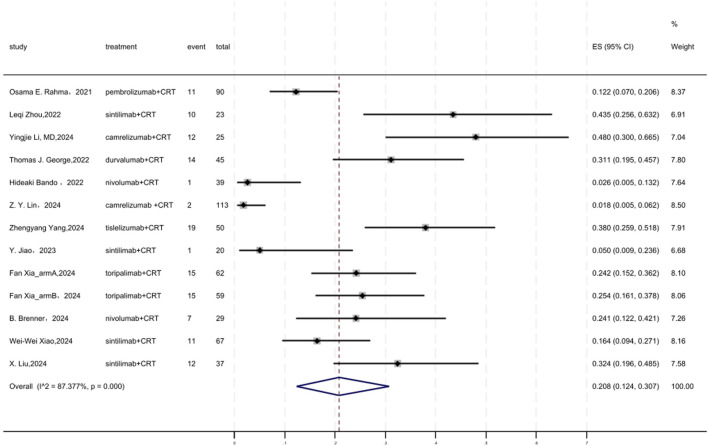
Forest plot of the meta-analysis for cCR.

Figure 3 displayed a forest plot of the meta-analysis focused on cCR. The cCR of neoadjuvant PD-(L)1 inhibitors combined with CRT was 0.208(95%CI = 0.124–0.307, I^2^ = 87.377%, P = 0.000). Results of the meta-analysis were shown in Table 2. Sensitivity analysis confirmed the stability of our findings (Supplementary Figure S3). Funnel plot assessment revealed no significant publication bias regarding cCR (Supplementary Figure S4).

### 3.5 MPR

**FIGURE 4:**
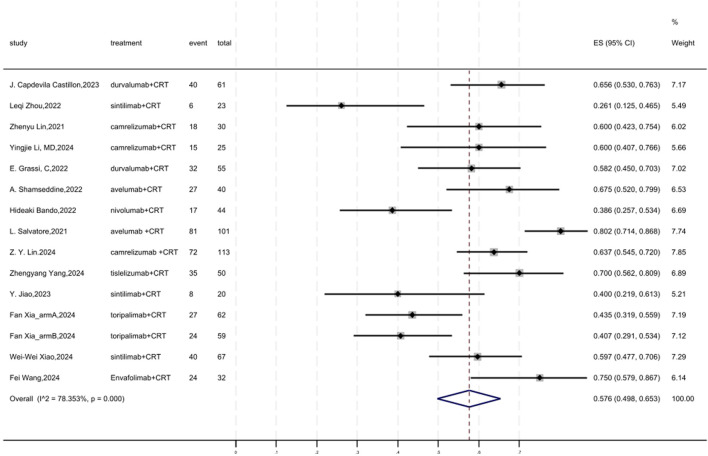
Forest plot of the meta-analysis for MPR.

Figure 4 displayed a forest plot of the meta-analysis focused on MPR. The overall pooled MPR of neoadjuvant PD-(L)1 inhibitors combined with CRT was 0.576(95%CI = 0.498–0.653, I^2^ = 78.353%, P = 0.000). Results of the meta-analysis were shown in Table 2. Sensitivity analysis confirmed the stability of our findings (Supplementary Figure S5). Funnel plot assessment revealed no significant publication bias regarding MPR (Supplementary Figure S6).

### 3.6 Surgery rate

**FIGURE 5:**
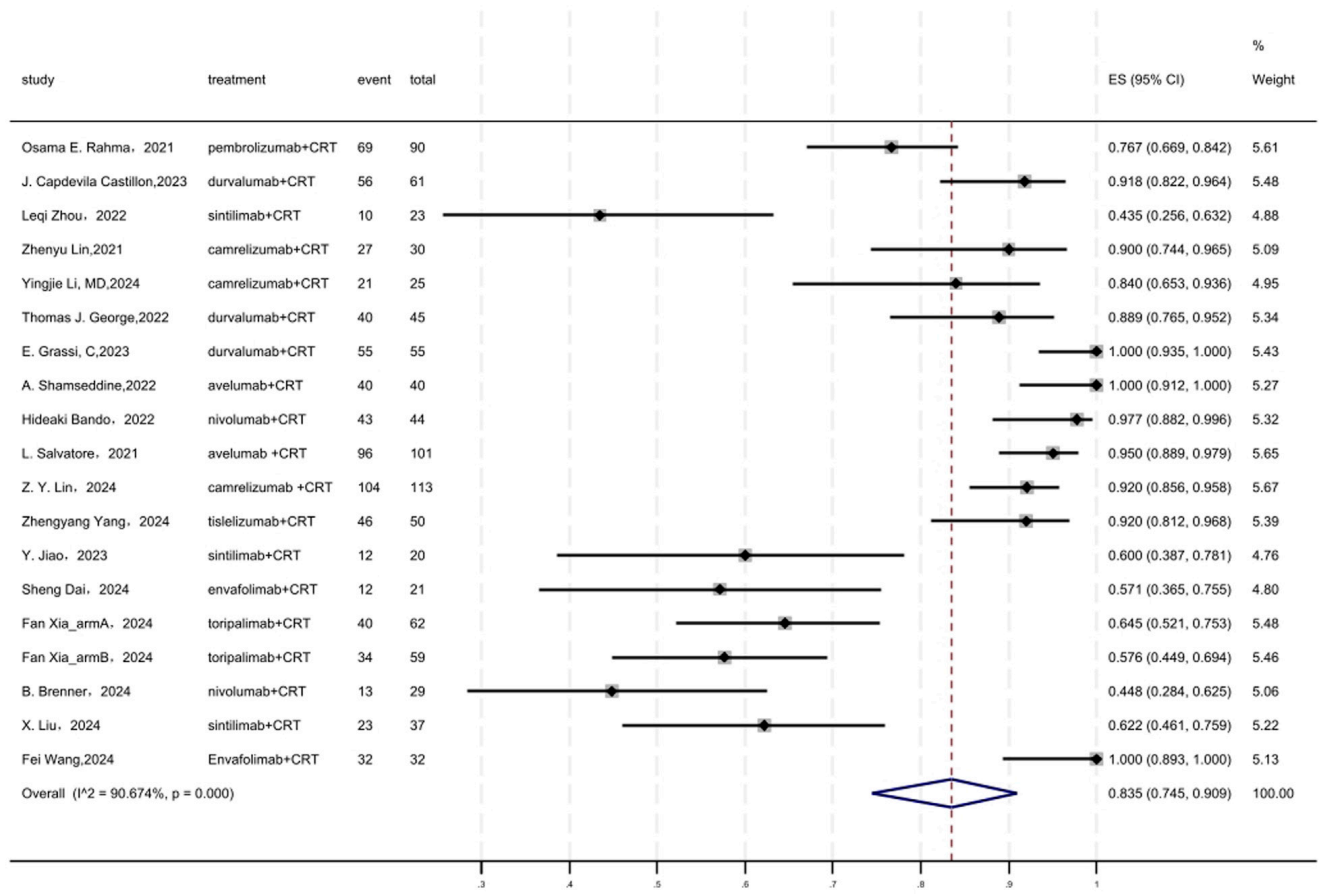
Forest plot of the meta-analysis for surgery rate.

Figure 5 displayed a forest plot of the meta-analysis focused on surgery rate. The surgery rate of neoadjuvant PD-(L)1 inhibitors combined with CRT was 0.835(95%CI = 0.745–0.909, I^2^ = 90.674%, P = 0.000). Results of the meta-analysis were shown in Table 2. Sensitivity analysis confirmed the stability of our findings (Supplementary Figure S7). Funnel plot assessment revealed no significant publication bias regarding surgery rate (Supplementary Figure S8).

### 3.7 R0 resection rate

**FIGURE 6:**
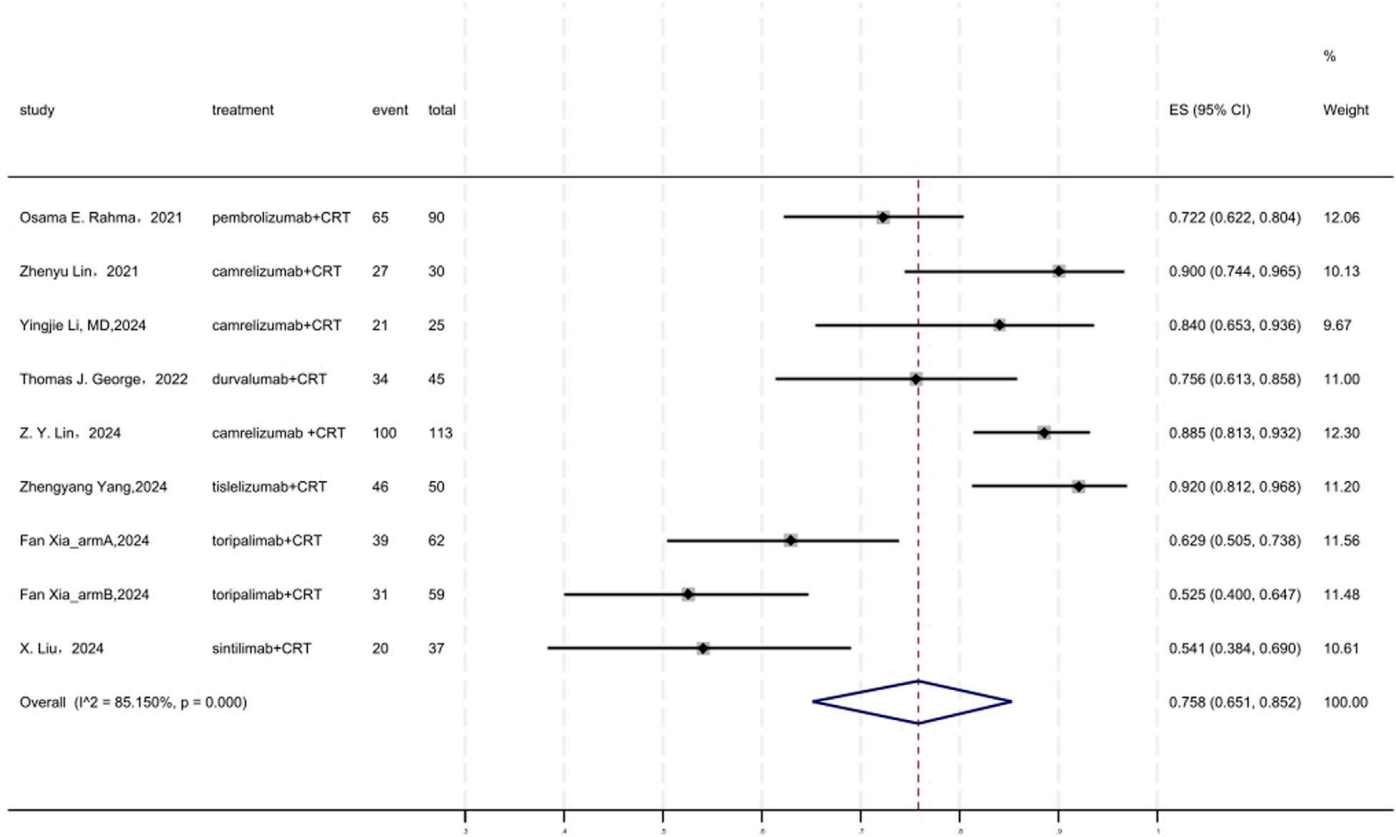
Forest plot of the meta-analysis for R0 resection rate.

Figure 6 displayed a forest plot of the meta-analysis focused on R0 resection rate. The R0 resection rate of neoadjuvant PD-(L)1 inhibitors combined with CRT was 0.758(95%CI = 0.651–0.852, I^2^ = 85.150%, P = 0.000). Results of the meta-analysis were shown in Table 2. Sensitivity analysis confirmed the stability of our findings (Supplementary Figure S9). Funnel plot assessment revealed no significant publication bias regarding R0 resection rate (Supplementary Figure S10).

### 3.8 SSS

**FIGURE 7:**
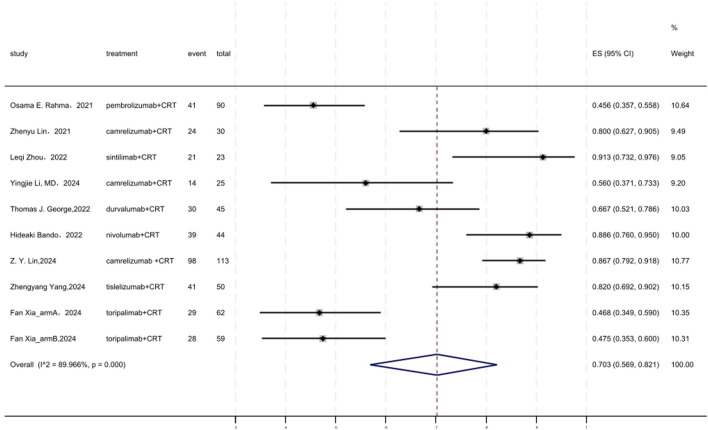
Forest plot of the meta-analysis for SSS.

Figure 7 displayed a forest plot of the meta-analysis focused on SSS. The SSS of neoadjuvant PD-(L)1 inhibitors combined with CRT was 0.703(95%CI = 0.569–0.821, I^2^ = 89.966%, P = 0.000). Results of the meta-analysis were shown in Table 2. Sensitivity analysis confirmed the stability of our findings (Supplementary Figure S11). Funnel plot assessment revealed no significant publication bias regarding SSS (Supplementary Figure S12).

### 3.9 3 years DFS

**FIGURE 8:**
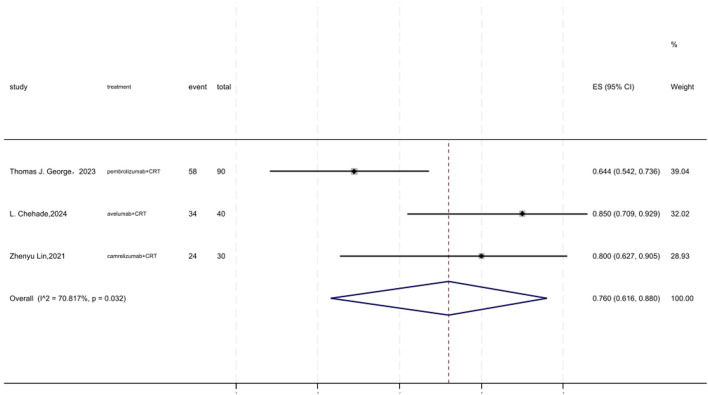
Forest plot of the meta-analysis for 3 years DFS.

Figure 8 displayed a forest plot of the meta-analysis focused on 3 years DFS. The 3 years DFS of neoadjuvant PD-(L)1 inhibitors combined with CRT was 0.760(95%CI = 0.616–0.880, I^2^ = 70.817%, P = 0.032). Results of the meta-analysis were shown in Table 2. Sensitivity analysis confirmed the stability of our findings (Supplementary Figure S13). Funnel plot assessment revealed no significant publication bias regarding DFS (Supplementary Figure S14).

### 3.10 Safety

**FIGURE 9:**
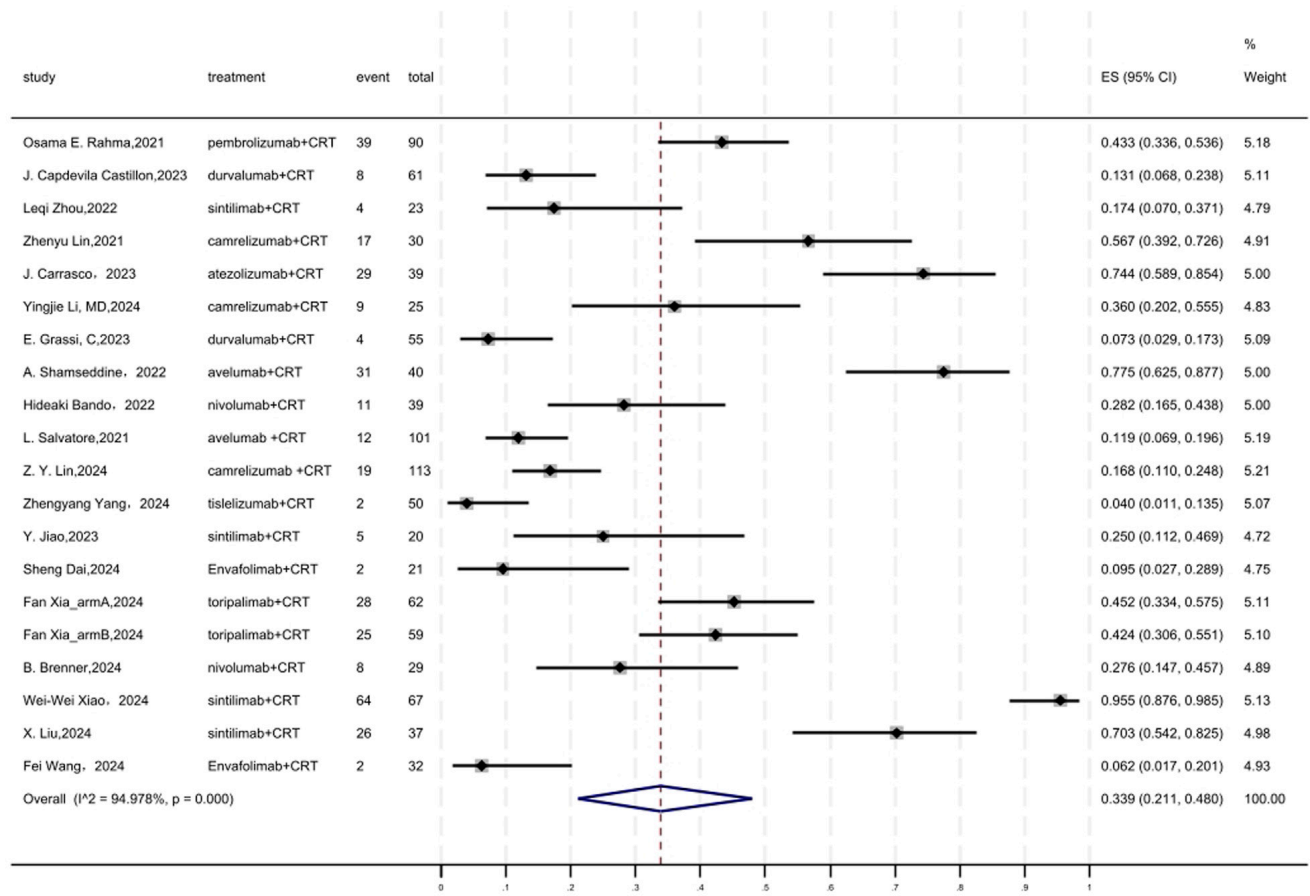
Forest plot of the meta-analysis of the meta-analysis for Grade ≥3 AEs.

Regarding safety, Grade ≥3 AEs rate was evaluated, which was reported in a total of 19 cohorts (Figure 9). The overall pooled result of Grade ≥3 AEs rate of neoadjuvant PD-(L)1 inhibitors combined with CRT was 0.339(95%CI = 0.211–0.480, I^2^ = 94.978%, P = 0.000). Results of the meta-analysis were shown in Table 2. Sensitivity analysis confirmed the stability of our findings (Supplementary Figure S15). Funnel plot assessment revealed moderate asymmetry in Grade ≥3 AE rates (Supplementary Figure S16).

### 3.11 Subgroup analysis

#### 3.11.1 Subgroup based on mismatch repair status

Subgroup analysis revealed pCR rates of 0.502 (95%CI = 0.103–0.900, I^2^ = 31.495%, P = 0.211) and 0.322 (95%CI = 0.263–0.385, I^2^ = 58.052%, P = 0.003) for the dMMR/MSI-H and pMMR/MSS subgroups, respectively (Supplementary Figure S17). Furthermore, Supplementary Figure S18 demonstrated that the subgroup analysis showed that the combined MPR rates for the dMMR/MSI-H and pMMR/MSS subgroups were 0.647 (95%CI = 0.233–0.977, I^2^ = 0.000%, P = 0.619) and 0.529 (95%CI = 0.429, 0.627, I^2^ = 74.874%, P = 0.000). Results of subgroup meta-analysis were shown in Table 3. The meta-regression showed no statistically significant differences between subgroups (Supplementary Figures S19, 20).

**TABLE 3:** Results of subgroup meta-analysis.

0utcomes	No. of studies	Heterogeneity		Overall effect size	95% CI of overall effect	Weight (%)
		I^2^ (%)	p-value			
pCR						
dMMR	5	31.495	0.211	0.502	0.103, 0.900	5.95
pMMR	14	58.052	0.003	0.322	0.263, 0.385	94.05
PD-L1 inhibitors	7	71.373	0.002	0.331	0.238, 0.430	30.54
PD-1 inhibitors	16	38.233	0.060	0.300	0.259, 0.343	69.46
SCRT	7	46.001	0.085	0.391	0.319, 0.466	32.55
LCRT	14	31.176	0.127	0.271	0.231, 0.313	67.45
concurrent immuno-CRT	11	31.494	0.147	0.308	0.266, 0.353	52.57
sequential immuno-CRT	12	64.730	0.001	0.301	0.232, 0.376	47.43
RCT	7	0.000	0.437	0.323	0.283, 0.365	37.26
single-arm	16	61.129	0.001	0.300	0.242, 0.361	62.74
MPR						
dMMR	4	0.000	0.619	0.647	0.233, 0.977	7.89
pMMR	11	74.874	0.000	0.529	0.429, 0.627	82.11
Grade ≥ 3 AEs						
PD-L1 inhibitors	5	93.869	0.000	0.206	0.044, 0.437	25.14
PD-1 inhibitors	15	94.727	0.000	0.388	0.235, 0.552	74.86

pCR: pathological complete response; dMMR: mismatch repair-deficient; pMMR:proficient mismatch repair; PDL-1, inhibitors: Programmed Death-Ligand 1 Inhibitor; PD-1, inhibitors: Programmed Cell Death Protein 1 Inhibitor; SCRT: short-course radiotherapy; LCRT: long-course radiotherapy; RCT: randomized controlled trial; MPR: major pathologic response; Grade ≥3 AEs: Grade≥3 adverse events.

However, the limited sample size in the dMMR/MSI-H subgroup could restrict the robustness of these results.

#### 3.11.2 Subgroup based on radiotherapy strategies

The subgroup analysis showed that the combined pCR rates for the long-course radiotherapy (LCRT) and short-course radiotherapy (SCRT) subgroups were 0.271 (95% CI = 0.231–0.313, I^2^ = 31.176%, P = 0.127) and 0.391 (95% CI = 0.319–0.466, I^2^ = 46.001%, P = 0.085) respectively (Supplementary Figure S21). Results of subgroup meta-analysis were shown in Table 3. The meta-regression showed no statistically significant differences between subgroups (Supplementary Figure S22).

#### 3.11.3 Subgroup based on neoadjuvant therapy strategies

Subgroup analysis revealed pCR rates of 0.308 (95%CI = 0.266–0.353, I^2^ = 31.494%, P = 0.147) and 0.301 (95%CI = 0.232–0.376, I^2^ = 64.730%, P = 0.001) for the concurrent and sequential immuno-CRT subgroups, respectively (Supplementary Figure S23). Results of subgroup meta-analysis were shown in Table 3. The meta-regression showed no statistically significant differences between subgroups (Supplementary Figure S24).

#### 3.11.4 Subgroup based on PD-1/PD-L1 inhibitors

Subgroup analysis revealed pCR rates of 0.331 (95%CI = 0.238–0.430, I^2^ = 71.373%, P = 0.002) and 0.300 (95%CI = 0.259–0.343, I^2^ = 38.233%, P = 0.060) for the PD-L1 and PD-1 inhibitors subgroups, respectively (Supplementary Figure S25). The safety profile of PD-L1 and PD-1 inhibitors was assessed, and the adverse effects grade≥3 were found to be 0.206 (95%CI = 0.044–0.437, I^2^ = 93.869%, P = 0.000) and 0.388 (95%CI = 0.235–0.552, I^2^ = 94.727%, P = 0.000) (Supplementary Figure S26). Results of subgroup meta-analysis were shown in Table 3. The meta-regression showed no statistically significant differences between subgroups (Supplementary Figures S27, 28). Grade ≥3 adverse events occurring frequently with PD-1 inhibitors included anemia (9%), diarrhea (5%), dermatitis (3%), AST elevation (2%), and colitis (1%), whereas PD-L1 inhibitors were predominantly associated with thrombocytopenia (3%), diarrhea (3%), abnormal liver function (3%), lipase/amylase elevations (2%), and increased AST/ALT (2%).

#### 3.11.5 Subgroup based on type of trials

Subgroup analysis revealed pCR rates of 0.323 (95%CI = 0.283–0.365, I^2^ = 0.000%, P = 0.437) and 0.300 (95%CI = 0.242–0.361, I^2^ = 61.129%, P = 0.001) for the RCT and single-arm subgroups, respectively (Supplementary Figure S29). Results of subgroup meta-analysis were shown in Table 3. The meta-regression showed no statistically significant differences between subgroups (Supplementary Figure S30).

## 4 Discussion

The major objectives of neoadjuvant therapy in LACRC are to accomplish complete removal of the tumour, reduce the likelihood of local recurrence and distant metastases by histological downstaging, and eliminate hidden micrometastatic disease with acceptable toxicity (Zhu et al., 2023). Obtaining a significant level of sphincter preservation is also actively pursued in LARC (Zhu et al., 2023). Advancements in achieving pCR or cCR and preserving organs appear to have been facilitated by the use of radiotherapy and various neoadjuvant polychemotherapy schemes in LARC (Zhu et al., 2023). The objective of our meta-analysis was to assess the safety and effectiveness of neoadjuvant PD-(L)1 inhibitors in patients with LARC by a systematic review and meta-analysis. The combined results of pCR, cCR, MPR, SSS, R0 resection rate, surgery rate, and 3-year DFS were 30.8%, 20.8%, 57.6%, 70.3%, 75.8%, 83.5%, and 76% respectively. Of all the adverse events, 33.9% were classified as Grade ≥3.

The NECTAR trial is the first documented multi-center study that examines the use of PD-1 blocked + CRT as neoadjuvant therapy for proficient mismatch repair (pMMR) or microsatellite stable (MSS) LARC patients by our knowledge (Yang et al., 2024c). The primary endpoint of pCR rates in this single-arm, non-randomized research was 40.0%, significantly greater than the 10%–20% rates achieved by conventional neoadjuvant treatments (Yen et al., 2021; Thommen and Schumacher, 2018). Following therapy, the levels of FR + CTCs, which are associated with the prognosis of cancer patients, showed a substantial reduction (Yang et al., 2024c). Furthermore, the combination was well tolerated without any unforeseen or novel safety adverse effects (Yang et al., 2024c). The fundamental components of the antitumor immune response are immunological clearance, immune homeostasis, and immune evasion (Yang et al., 2022). The immunological checkpoints PD-1 and PD-L1 are essential components that function as inhibitory signals on the immune system and are pivotal in the process of tumour immunological evasion (Bailey and Maus, 2019). Tumour cells exploit the detection of the T-cell receptor following the interaction of PD-1 and PD-L1, hence further inhibiting immunity and eluding immune monitoring (He et al., 2021). The first report in 2002 indicated that the PD-1 pathway, which mediates tumour immunity, is characterised by the weakening of T cell cytolytic activity and subsequent considerable promotion of tumour development and invasion when PD-L1 is overexpressed (Iwai et al., 2002). Curiously, the use of monoclonal antibodies against PD-L1 could neutralise such effect (Wu et al., 2019). Anti-PD-1/PD-L1 therapy selectively binds to PD-1 and PD-L1 respectively, therefore inhibiting the co-localization of PD-1 on T cell surfaces and PD-L1 on tumour cell surfaces (Bie et al., 2022). The implementation of such function has the potential to counteract the suppressive impact of tumour cells on the immune system and reinstate the antitumor immunity (Yang et al., 2022). PD-L1 interacts with PD-1, and this immune checkpoint pathway plays a crucial role in suppressing the T cell-mediated antitumor immune response by modulating the interaction between PD-1 and CD80 on T cells in the TME (Zhang et al., 2022). PD-1 and PD-L1 inhibitors suppress the function of this immune checkpoint, therefore “releasing the immune brake” in the tumour microenvironment by stimulating T cell activation, reengaging the immune response of T cells against tumours, and enhancing immune system activity (Sahin et al., 2023; Rahib et al., 2021). Significant upregulation of PD-L1 in tumour nests was shown to be strongly linked to positive results following neoadjuvant chemotherapy and chemoradiotherapy (Zhang SY. et al., 2019; Chen et al., 2019). Preclinical data indicates that the combination of radiotherapy with PD-1/PD-L1 antibodies can modify the tumour microenvironment, counteract immunosuppression, increase the produce of antitumor cytokines by T-cells, and improve the effectiveness of radiotherapy (Dovedi and Illidge, 2015; Dovedi et al., 2014).

Our meta-analysis revealed a pCR rate of 0.308, which is somewhat modest. Possible contributing reasons to this outcome include the existence of resistant subgroups among patients with LARC. Elaborate mechanisms underlie resistance to ICIs therapy (Zhu et al., 2023). Investigation revealed that genetic modifications exerted an impact on the several stages of immune activation (Zhu et al., 2023). Impairment of immune recognition caused by alterations in the WNT signalling system (Zhu et al., 2023). A genetic mutation resulting in a biallelic loss of b2 microglobulin (b2M), a component of MHC class I, and single-copy loss events in HLA molecules cause abnormalities in the presentation of antigens (Zhu et al., 2023). Mutations in genes associated to immune response, namely, those involved in T-cell responses, B-cell development, and NK cell function, have been implicated in the decline of immunological function (Zhu et al., 2023). These possible alterations in specific immune pathways may lead to the development of immunological tolerance and inherent resistance to ICIs therapy (Chalabi et al., 2020). The IFN-g signalling pathway was identified as a crucial factor in the development of immunotherapy resistance among the several mechanisms (Zhu et al., 2023). T cell-initiated IFN-g signalling can stimulate the production of PD-L1 via JAK-STAT signaling (Zhu et al., 2023).

Therefore, it is crucial to carefully evaluate the recipients of this neoadjuvant approach (Gao et al., 2023). The VOLTAGE-A trial shown that individuals with higher levels of PD-L1 expression and CD8/eTreg ratio before to treatment were more likely to experience enhanced immunotherapy outcomes (Gao et al., 2023). Out of patients with PD-L1 (TPS) ≥ 1%, 75% of them attained pCR, but in the PD-L1 (TPS) < 1% group, only 17% of patients obtained pCR (Bando et al., 2022b). Through the analysis of clinical characteristics, we determined that CEA was not a reliable indicator of tumour response (Gao et al., 2023). The prevalence of pCR was 11.1% in individuals with CEA elevation, compared to 70.6% in those without CEA elevation (Gao et al., 2023). Consistent with prior research, pre-treatment CEA was found to be inversely associated with pCR. (Lee et al., 2015; Das et al., 2007). Another predicted criterion found was being under 50 years old (Gao et al., 2023). A 100% (4/4) pCR rate is seen in these young-onset rectal patients following neoadjuvant therapy (Gao et al., 2023). Colorectal patients under 50 had limited anti-tumor immune responses due to inadequate tumour differentiation and low tumor-infiltrating lymphocytes (Ugai et al., 2022). Nevertheless, this state can be inverted by the combination of chemoradiotherapy and immunotherapy (Gao et al., 2023).

Subgroup analyses were performed for dMMR/MSI-H, LCRT, SCRT, PD-1, PD-L1, and concurrent and sequential immuno-chemoradiotherapy. Our study found 27.1% and 39.1% pCR rates for PD-1/PD-L1 inhibitors with LCRT and SCRT. SCRT with immunotherapy had significantly higher pCR rates than LCRT. In colorectal cancer, the study found that 8Gy × 3 was the most effective fractionation mode when combined with immunotherapy at three doses: 16.4Gy × 1, 8Gy × 3, and 2Gy × 18. It significantly increased tumor-infiltrating lymphocytes and PD-L1 and TIGIT expression. When paired with an ICI, it had the strongest tumour control (90% full response) (Grapin et al., 2019). Short-course hypofractionated radiation controls tumour growth and attracts T lymphocytes in draining lymph nodes while retaining suppressor and effector T cells. Meanwhile, short-course hypofractionated radiation increases IL-8, IL-6, TNFa, and other variables, promoting dendritic cell (DC) development and activation. Thus, this method improves immunotherapy (John-Aryankalayil et al., 2010; Kulzer et al., 2014).

In dMMR/MSI-H colorectal cancer, the ICIs appear favorable clinical benefits (Cercek et al., 2022), while in pMMR/MSS subsets, the slight efficacies of ICIs have been reported (Yang et al., 2022; Chalabi et al., 2020). Moreover, patients with MSI-H only account for about 5%, and the majority CRC population is MSS (Chalabi et al., 2020). Improving the immunotherapeutic sensitivity of MSS patients remains a challenge (Wang YQ. et al., 2022). There is growing evidence suggesting that immune checkpoint inhibitors are highly effective for the treatment of mismatch-repair deficient or microsatellite instability-high colorectal cancers (Robert et al., 2015; Overman et al., 2017; Le et al., 2020; André et al., 2020b). Based on data from the phase 3 KEYNOTE-177 trial, anti-PD-1 therapy has been recommended as the standard first-line treatment for mismatch-repair deficient metastatic colorectal cancer (André et al., 2020b). Immune checkpoint inhibitors have also shown activity in patients with non-metastatic mismatch-repair deficient colorectal cancer (Chen et al., 2023). In the NICHE-2 study, 95% of patients with locally advanced mismatch-repair deficient colon cancers had a major pathological response, with 67% showing a pathological complete response (Wang et al., 2020).

A previous meta-analysis revealed pCR of 100% in the MSIH/dMMR LARC subgroup (Yang L. et al., 2024), while our result revealed that the pCR rate of the MSIH/dMMR LARC subgroup was only 50.2%. The reason is the differences in the number of studies included between two meta-analysis. Our analysis incorporated the highest quantity of research up until now, all of which were prospective studies. Specifically, Z.Y. Lin et al. reported that though three MSI-H patients with LARC responded to camrelizumab + CRT, none of the MSI-H patients achieved pCR (Lin et al., 2024). We failed to explore the effect of biomarker variability since the absence of detail baseline characteristics of these three MSI-H patients. The patient selection of this study was similar to those of other studies. The potential reason for the lower pCR rates observed might be the specific PD-1 inhibitor used (camrelizumab). Considering the small sample of MSI-H patients, additional research is necessary to examine the effectiveness and safety of this experimental therapy in the MSI-H patients.

Furthermore, a meta-analysis revealed that PD-1 inhibitors (40%) and sequential immuno-CRT (40%) had been associated to greater rates pCR compared to PD-L1 inhibitors (32%) and concurrent immune CRT (30%) (Yang L. et al., 2024). We found little difference in pCR rates across PD-1 inhibitors (30%), PD-L1 inhibitors (33.1%), sequential immuno-CRT (30.1%), and concurrent immuno-CRT (30.8%).In PD mice, PD-1 inhibitors disrupted the interaction of PD-1/PD-L2 with other partners, such as repulsive guidance molecule b (RGMb), resulting in undesirable results (Xiao et al., 2014). This can be useful for medical practitioners.

In addition, PD-(L)1 inhibitors in neoadjuvant therapy may cause negative effects. Our thorough research showed that this medication did not significantly increase serious AEs. The total anal preservation rate was 75.8%, with a grade ≥3 toxicity level of 33.9%. The high anal preservation rate improved patient quality of life. The research found that nIT reduced anal sphincter dysfunction risk compared to nCT and nCRT (Zhang et al., 2019c; Wensink et al., 2021). Clinical trials show that grade ≥3 immune-related adverse events (irAEs) varied from 13% to 22% with ICI alone and 22%–64% with dual ICIs (Hirano et al., 2021). In KEYNOTE 177, the adverse event rate was 22% (33/153), with 9% (14/153) grade ≥3 in the pembrolizumab group, 13% (18/143), and 2% (3/143) in the chemotherapy group (Diaz et al., 2022). Furthermore, there is data that substantiates the notion that individual PD-1/PD-L1 inhibitors result in a lower incidence of treatment-related side events compared to chemotherapy applied alone (André et al., 2020a). The findings showed that the PD-1 subgroup had 38.8% more adverse events than the PD-L1 subgroup (20.6%), which can be attributed to their broader immunological activity stemming from distinct mechanistic differences: PD-1 inhibitors directly block the PD-1 receptor, disrupting its interactions with both PD-L1 and PD-L2 ligands, thus eliciting more extensive immune activation, particularly in tissues with elevated PD-L2 expression; in contrast, PD-L1 inhibitors selectively interrupt the PD-1/PD-L1 axis without affecting PD-1/PD-L2 interactions, resulting in a narrower spectrum of immune activation and comparatively lower toxicity (Brahmer et al., 2018b; Wang et al., 2019). Furthermore, given the widespread expression of PD-1 receptors across multiple immune cell types, including T cells, B cells, and NK cells, PD-1 blockade induces broader activation of diverse immune cell subsets, whereas PD-L1 expression is predominantly restricted to tumor cells and certain immune cell populations, limiting the immunological impact and associated adverse effects of PD-L1 inhibitors (Wang et al., 2019; Chen and Mellman, 2017). Several academic organisations, such as the European Society for Medical Oncology, American Society of Clinical Oncology, and NCCN, have developed criteria and guidelines for irAEs that might accompany their clinical usage (Haanen et al., 2017; Brahmer et al., 2018a; Thompson et al., 2020). These irAEs induced by PD-1/PD-L1 inhibitors are generally tolerable, predictable, and manageable (Yang et al., 2022). According to NCCN, ASCO/SITC, and ESMO guidelines (Schneider et al., 2023; Schneider et al., 2021; Haanen et al., 2022), management of grade ≥3 adverse events during PD-1/PD-L1 therapy for locally advanced rectal cancer requires a systematic approach. For immune-related colitis/diarrhea, withhold immunotherapy and administer methylprednisolone 1–2 mg/kg/day IV, with infliximab for steroid-refractory cases. Hepatic toxicity management includes treatment interruption, corticosteroids, and mycophenolate mofetil for inadequate response. Hematologic toxicities necessitate immunotherapy interruption, chemoradiotherapy modification, close monitoring. Severe anemia or thrombocytopenia may require transfusions or hematopoietic growth factor support. Resume treatment upon toxicity improvement to grade ≤1, considering permanent discontinuation for life-threatening events. Supplementary Table S1 listed the common AEs and their management.

The current study had several strengths. Firstly, limited meta-analyses have evaluated the efficacy and safety of neoadjuvant PD-1/PD-L1 inhibitors plus CRT for LARC. We performed a systematic review and meta-analysis incorporating the most recent studies on neoadjuvant PD-1/PD-L1 inhibitors plus CRT for LARC. The large sample size enhanced statistical power, precision, and reliability of the pooled estimates. Second, the predominantly high methodological quality of included studies, especially the uniformly high-quality RCTs, substantially supports the robustness and credibility of the meta-analysis findings. Third, the outcomes were aggregated using subgroup analysis because to variations in the literature, including of dMMR/MSI-H and pMMR/MSS subgroups, LCRT and SCRT subgroups, the concurrent and sequential immuno-chemoradiotherapy subgroups, as well as PD-L1 and PD-1 inhibitor subgroups. Additionally, the comprehensive subgroup analyses performed in this study effectively identified potential sources of heterogeneity, thereby improving the robustness and clinical interpretability of the results. These methodological advantages facilitated evidence-based clinical decision-making and provided valuable insights for future research directions.

Nonetheless, our study exhibited several limitations. First, the inclusion of multiple single-arm studies introduced inherent selection bias and confounding variables due to the absence of randomization and control groups, potentially overestimating treatment effects. Significant statistical heterogeneity (high I^2^ values) persisted despite random-effects modeling, stemming from variability in patient populations, intervention protocols, and outcome definitions, thus reducing the precision of pooled estimates. Second, the predominance of short-term follow-up data precluded definitive conclusions regarding sustained efficacy and long-term safety profiles, particularly problematic for chronic conditions requiring extended management. Third, the small number of studiesand patients in the dMMR/MSI-H subgroup significantly impacted the generalizability and reliability of findings, including reduced statistical power, heightened vulnerability to random variations. Additional constraints include potential publication bias and inconsistent outcome measurement methodologies across studies. Therefore, multicenter larger-scale RCTs with extended follow-up periods are needed to confirm our findings. It is advised to explore the optimal radiotherapy sequencing, the optimal PD-1/PD-L1 inhibitor and the effect of biomarker variability (e.g., PD-L1 expression levels). It is suggested to include more patients with dMMR/MSI-H to evaluate the efficacy of PD-1/L1 inhibitors plus CRT in this subgroup patients.

In Conclusion, this meta-analysis demonstrated that neoadjuvant PD-1/PD-L1 inhibitors combined with CRT could improve MPR and pCR rates in pMMR/MSS locally advanced rectal cancer, while patients with dMMR/MSI-H tumors exhibited notably high pathological responses. Moreover, it provided favorable clinical outcomes, including enhanced anal preservation rates, increased R0 resection rates, and promising 3 years DFS. Subgroup analyses further indicated superior pCR rates associated with short-course radiotherapy, PD-1 inhibitors, and sequential immunotherapy compared to long-course radiotherapy, PD-L1 inhibitors, and concurrent immunotherapy. Collectively, these findings supported the clinical utility of neoadjuvant PD-1/PD-L1 inhibitors combined with CRT in LARC.

## Data Availability

The datasets presented in this study can be found in online repositories. The names of the repository/repositories and accession number(s) can be found in the article/Supplementary Material.
