# Supplementary material for: Efficacy and safety of neoadjuvant PD-1 inhibitors or PD-L1 inhibitors combined with chemoradiotherapy for locally advanced rectal cancer: a systematic review and meta-analysis
Source: Front Pharmacol. 2025 May 16;16:1570467. doi: 10.3389/fphar.2025.1570467 (PMC12122451; doi:10.3389/fphar.2025.1570467)
Supplement: Supplementary file 1 [file DataSheet2.pdf]

Quality assessment for RCTS using Jadad Scale

Author	Study described as randomized: Yes = 1 point, No = 0 points	Method of randomization described and appropriate: Yes = add 1 point	Method inappropriate: Deduct 1 point	Study described as double-blind: Yes = 1 point, No = 0 points	Method of blinding described and appropriate: Yes = add 1 point	Method inappropriate: Deduct 1 point	Description of withdrawals and dropouts: Yes = 1 point, No = 0 points	Jadad Scale
Osama E.Rahma(2021)[24]	1	1	0	1	1	0	1	5
T. Zhang(2023)[35]	1	1	0	1	1	0	0	4
Fan Xia(2024)[39]	1	1	0	1	1	0	1	5
Yingchi Yang(2024)[41]	1	1	0	1	1	0	1	5
Wei-Wei Xiao(2024)[42]	1	1	0	1	1	0	1	5

Quality assessment for non-RCTS using MINORS

Author	A clearly stated aim (0/1/2)	Inclusion of consecutive patients (0/1/2)	Prospective collection of data (0/1/2)	Endpoints appropriate to the aim of the study (0/1/2)	Unbiased assessment of the study endpoint (0/1/2)	Follow-up period appropriate to the aim of the study (0/1/2)	Loss to follow-up less than 5% (0/1/2)	Prospective calculation of the study size (0/1/2)	MINORS score
J.Capdevila Castillon(2023)[25]	2	2	2	2	2	0	0	2	12
Leqi Zhou(2022)[26]	2	2	2	2	2	0	2	2	14
Zhenyu Lin(2021)[27]	2	2	2	2	2	0	2	2	14
J. Carrasco(2023)[28]	2	2	2	2	2	0	1	2	13
Yingjie Li(2024)[29]	2	2	2	2	2	1	0	2	13
Thomas J.George(2022)[30]	2	2	2	2	2	0	0	2	12
E. Grassi, C(2023)[31]	2	2	2	2	2	2	0	2	14
A.Shamseddine(2022)[32]	2	2	2	2	2	2	0	2	14
Hideaki Bando(2022)[33]	2	2	2	2	2	1	2	2	15
L. Salvatore(2021)[34]	2	2	2	2	2	0	1	2	13
Jiale Gao(2023)[36]	2	2	2	2	2	2	0	2	14
Y. Jiao(2023)[37]	2	2	2	2	2	0	0	2	12
Sheng Dai(2024)[38]	2	2	2	2	2	0	1	2	13
B. Brenner(2024)[40]	2	2	2	2	2	1	0	2	13
X. Liu(2024)[43]	2	2	2	2	2	0	1	2	13
Fei Wang(2024)[44]	2	2	2	2	2	2	0	2	14
