# Supplementary material for: Efficacy and safety of neoadjuvant PD-1 inhibitors or PD-L1 inhibitors combined with chemoradiotherapy for locally advanced rectal cancer: a systematic review and meta-analysis
Source: Front Pharmacol. 2025 May 16;16:1570467. doi: 10.3389/fphar.2025.1570467 (PMC12122451; doi:10.3389/fphar.2025.1570467)
Supplement: Supplementary file 2 [file Table1.pdf]

**Supplement Table 1** Common AEs and opposite management.

| Common AEs | Management |
|---|---|
| anemia | Grade 1-2: Oral iron supplementation, nutritional support<br>Grade ≥3: 1)Temporarily hold immunotherapy and possibly chemoradiotherapy; 2)Red blood cell transfusions for symptomatic patients or Hb <7.0 g/dL; 3)Consider erythropoiesis-stimulating agents if not contraindicated; 4)For immune-mediated anemia: prednisone 1-2 mg/kg/day |
| diarrhea | Grade 1-2: 1)Antidiarrheal medications (loperamide); 2)Hydration, electrolyte replacement; 3)Low-residue diet;<br>Grade ≥3: 1)NPO or clear liquid diet initially; 2)Systemic corticosteroids (prednisone 1-2 mg/kg/day); 3)IV fluids and electrolyte correction; 4)Stool studies to rule out infection; 5)For steroid-refractory cases: consider infliximab |
| colitis | Grade 1: Symptomatic treatment, monitor closely<br>Grade 2: 1)Oral corticosteroids (prednisone 0.5-1 mg/kg/day); 2)Antidiarrheal medications if infectious causes excluded<br>Grade 3-4: 1)Hospital admission;2)IV methylprednisolone 1-2 mg/kg/day; 3)For steroid-refractory cases: infliximab 5-10 mg/kg; 4)Consider vedolizumab for recurrent episodes |
| AST/ALT increase | Grade 1: Monitor LFTs weekly until stabilization<br>Grade 2: 1)Monitor LFTs every 3-5 days; 2)Consider temporary interruption of immunotherapy; 3)Observe for resolution or progression<br>Grade ⩾3: 1)Initiate prednisone 1-2 mg/kg/day (or equivalent); 2)Hepatology consultation; 3)If no improvement within 3-5 days, consider mycophenolate mofetil; 4)Avoid infliximab due to potential hepatotoxicity |
| dermatitis | Grade 1: 1)Topical emollients and moisturizers; 2)Low-potency topical corticosteroids for pruritus; 3)Oral antihistamines for symptomatic relief<br>Grade 2: 1)Medium to high-potency topical corticosteroids; 2)Consider temporary interruption of immunotherapy; 3)Dermatology consultation<br>Grade 3: 1)Interrupt immunotherapy; 2)Systemic corticosteroids (prednisone 0.5-1 mg/kg/day); 3)Consider reducing radiation dose if radiation dermatitis component<br>Grade 4: 1)Hospitalize immediately; 2)High-dose systemic corticosteroids (methylprednisolone 1-2 mg/kg/day); 3)Urgent dermatology and burn unit consultation |
| thrombocytopenia | Grade 1: 1)Monitor platelet counts weekly; 2)No specific intervention needed<br>Grade 2: 1)Modify or temporarily interrupt chemotherapy; 2)Monitor platelet counts twice weekly; 3)Consider growth factor support if neutropenia also present |

| | |
|---|---|
| | Grade 3: 1)Interrupt immunotherapy and chemoradiotherapy; 2)If immune-mediated: prednisone 1-2 mg/kg/day; 3)Consider radiotherapy dose modification; 4)Hematology consultation<br>Grade 4: 1)Discontinue immunotherapy temporarily; 2)Platelet transfusions for active bleeding or counts <10,000/μL; 3)High-dose corticosteroids if immune-mediated; 4)Consider IVIG for severe immune thrombocytopenia; 5)Avoid invasive procedures |
| liver function abnormality | Grade 1: 1)Monitor LFTs weekly; 2)Continue treatment with close surveillance<br>Grade 2: 1)Consider temporary interruption of immunotherapy; 2)Monitor LFTs every 3-5 days; 3)If persistent >1 week or worsening, initiate prednisone 0.5-1 mg/kg/day;<br>Grade 3: 1)Interrupt immunotherapy and chemoradiotherapy; 2)Initiate prednisone 1-2 mg/kg/day or methylprednisolone equivalent; 3)Hepatology consultation; 4)If no improvement within 2-3 days, consider mycophenolate mofetil (500-1000 mg twice daily)<br>Grade 4: 1)Permanently discontinue immunotherapy; 2)Hospitalize patient; 3)High-dose corticosteroids (methylprednisolone 2 mg/kg/day); 4)Consider mycophenolate mofetil early if severe; 5)Avoid infliximab due to potential hepatotoxicity |
| lipase/amylase increase | Grade 1 (asymptomatic): 1)Continue treatment with close monitoring; 2)Weekly lipase/amylase measurements; 3)Low-fat diet recommendation<br>Grade 2 (asymptomatic): 1)Consider temporary interruption of immunotherapy; 2)Monitor enzymes every 3-5 days; 3)Gastroenterology consultation if persistent elevation<br>Grade 3 (with or without symptoms): 1)Interrupt immunotherapy and possibly chemoradiotherapy; 2)NPO if symptomatic; 3)IV hydration if indicated; 4)Initiate prednisone 0.5-1 mg/kg/day if immune-mediated pancreatitis suspected; 5)Gastroenterology consultation<br>Grade 4 or symptomatic pancreatitis: 1)Discontinue immunotherapy; 2)Hospital admission; 3)NPO, IV hydration, pain management; 4)Methylprednisolone 1-2 mg/kg/day IV; 5)Consider TPN if prolonged NPO status necessary |
