# Supplementary material for: Efficacy and safety of neoadjuvant PD-1 inhibitors or PD-L1 inhibitors combined with chemoradiotherapy for locally advanced rectal cancer: a systematic review and meta-analysis
Source: Front Pharmacol. 2025 May 16;16:1570467. doi: 10.3389/fphar.2025.1570467 (PMC12122451; doi:10.3389/fphar.2025.1570467)
Supplement: Supplementary file 3 [file DataSheet1.pdf]

PubMed		
No.	Query	Results
1	(((PD-1 inhibitor[Title/Abstract]) OR (Pembrolizumab[Title/Abstract])) OR (SCH-900475[Title/Abstract])) OR (Lambrolizumab[Title/Abstract])) OR (MK-3475[Title/Abstract])) OR (Keytruda[Title/Abstract])) OR (Nivolumab[Title/Abstract])) OR (Opdivo[Title/Abstract])) OR (ONO-4538[Title/Abstract])) OR (ONO 4538[Title/Abstract])) OR (ONO4538[Title/Abstract])) OR (MDX-1106[Title/Abstract])) OR (MDX 1106[Title/Abstract])) OR (MDX1106[Title/Abstract])) OR (BMS-936558[Title/Abstract])) OR (BMS 936558[Title/Abstract])) OR (BMS936558[Title/Abstract])) OR (Toripalimab[Title/Abstract])) OR (Tislelizumab[Title/Abstract])) OR (BGB-A317[Title/Abstract])) OR (Camrelizumab[Title/Abstract])) OR (carrelizumab[Title/Abstract])) OR (SHR-1210[Title/Abstract])) OR (SHR 1210[Title/Abstract])) OR (GLS-010[Title/Abstract])) OR (Cemiplimab[Title/Abstract])) OR (REGN2810[Title/Abstract])) OR (Sintilimab[Title/Abstract])) OR (IBI308[Title/Abstract])) OR (IBI-308[Title/Abstract])) OR (Zimberelimab[Title/Abstract])) OR (Prolgolimab[Title/Abstract])) OR (Dostarlimab[Title/Abstract])) OR (Jemperli[Title/Abstract])) OR (dostarlimab-gxly[Title/Abstract])) OR (TSR-042[Title/Abstract])) OR (GSK4057190[Title/Abstract])) OR (PD-L1 inhibitor[Title/Abstract])) OR (Atezolizumab[Title/Abstract])) OR (anti-PDL1[Title/Abstract])) OR (immunoglobulin G1, anti-(human CD antigen CD274) (human monoclonal MDPL3280a heavy chain), disulfide with human monoclonal MDPL3280a kappa-chain, dimer[Title/Abstract])) OR (MPDL3280A[Title/Abstract])) OR (MPDL-3280A[Title/Abstract])) OR (Tecentriq[Title/Abstract])) OR (RG7446[Title/Abstract])) OR (RG-7446[Title/Abstract])) OR (Durvalumab[Title/Abstract])) OR (MEDI4736[Title/Abstract])) OR (MEDI-4736[Title/Abstract])) OR (Imfinzi[Title/Abstract])) OR (Avelumab[Title/Abstract])) OR (MSB-0010682[Title/Abstract])) OR (MSB0010682[Title/Abstract])) OR (bavencio[Tite/Abstract])) OR (MSB0010718C[Title/Abstract])) OR (MSB-0010718C[Title/Abstract])) OR (PD-1 Inhibitors[Title/Abstract])) OR (PD 1 Inhibitors[Title/Abstract])) OR (PD-1 Inhibitor[Title/Abstract])) OR (Inhibitor, PD-1[Title/Abstract])) OR (PD 1 Inhibitor[Title/Abstract])) OR (Programmed Cell Death Protein 1 Inhibitor[Title/Abstract])) OR (Programmed Cell Death Protein 1 Inhibitors[Title/Abstract])) OR (PD-L1 Inhibitors[Title/Abstract])) OR (PD L1 Inhibitors[Title/Abstract])) OR (PD-L1 Inhibitor[Title/Abstract])) OR (PD L1 Inhibitor[Title/Abstract])) OR (Programmed Death-Ligand 1 Inhibitors[Title/Abstract])) OR (Programmed Death Ligand 1 Inhibitors[Title/Abstract])) OR (PD-1-PD-L1 Blockade[Title/Abstract])) OR (Blockade, PD-1-PD-L1[Title/Abstract])) OR (PD 1 PD L1 Blockade[Title/Abstract])	23555 results
2	Rectal Neoplasm[Title/Abstract] OR Rectal Neoplasms[Title/Abstract] OR Rectum Neoplasm[Title/Abstract] OR Rectum Neoplasms[Title/Abstract] OR Neoplasm, Rectum[Title/Abstract] OR Neoplasm, Rectal[Title/Abstract] OR Neoplasms, Rectal[Title/Abstract] OR Neoplasms, Rectum[Title/Abstract] OR Neoplasm of Rectum[Title/Abstract] OR Neoplasm of the Rectum[Title/Abstract] OR Rectum	61,486 results

	Cancer[Title/Abstract] OR Rectum Cancers[Title/Abstract] OR Rectal Cancer[Title/Abstract] OR Rectal Cancers[Title/Abstract] OR Cancer, Rectal[Title/Abstract] OR Cancer, Rectum[Title/Abstract] OR Cancers, Rectal[Title/Abstract] OR Cancers, Rectum[Title/Abstract] OR Cancer of Rectum[Title/Abstract] OR Cancer of the Rectum[Title/Abstract] OR Rectal Tumors[Title/Abstract] OR Rectal Tumor[Title/Abstract] OR Rectum Tumor[Title/Abstract] OR Rectum Tumors[Title/Abstract] OR Tumor, Rectal[Title/Abstract] OR Tumors, Rectal[Title/Abstract] OR Tumor, Rectum[Title/Abstract] OR Tumors, Rectum[Title/Abstract] OR Tumor of Rectum[Title/Abstract] OR Tumor of the Rectum[Title/Abstract] OR Rectal Carcinoma[Title/Abstract] OR Rectal Carcinomas[Title/Abstract] OR Rectum Carcinoma[Title/Abstract] OR Rectum Carcinomas[Title/Abstract] OR Carcinoma, Rectal[Title/Abstract] OR Carcinomas, Recta[Title/Abstract] OR Carcinoma, Rectum[Title/Abstract] OR Carcinomas, Rectum[Title/Abstract] OR Carcinoma of Rectum[Title/Abstract] OR Carcinoma of the Rectum[Title/Abstract]	
3	Neoadjuvant	51299results
4	(((randomized controlled trial [pt] OR controlled clinical trial [pt] OR randomized [tiab] OR placebo [tiab] OR clinical trials as topic [mesh: noexp] OR randomly [tiab] OR trial [ti]) NOT (animals [mh] NOT hu mans [mh])) OR (Prospective[Title/Abstract])) OR (Retrospective[Title/Abstract])	1461947 1,46 1,947 results
5	1 AND 2 AND 3 AND 4	30 results

Web of science		
No.	Query	Results
1	((TS=(PD-1 inhibitor)) OR TS=(PD-1 Inhibitors)) OR TS=(PD 1 Inhibitors)) OR TS=(PD-1 Inhibitor)) OR TS=(Inhibitor, PD-1)) OR TS=(PD 1 Inhibitor)) OR TS=(Programmed Cell Death Protein 1 Inhibitor)) OR TS=(Programmed Cell Death Protein 1 Inhibitors)) OR TS=(Pembrolizumab)) OR TS=(SCH-900475)) OR TS=(Lambrolizumab)) OR TS=(MK-3475)) OR TS=(Keytruda)) OR TS=(Nivolumab)) OR TS=(Opdivo)) OR TS=(ONO-4538)) OR TS=(ONO 4538)) OR TS=(ONO4538)) OR TS=(MDX-1106)) OR TS=(MDX 1106)) OR TS=(MDX1106)) OR TS=(BMS-936558)) OR TS=(BMS 936558)) OR TS=(BMS936558)) OR TS=(Toripalimab)) OR TS=(Tislelizumab)) OR TS=(BGB-A317)) OR TS=(Camrelizumab)) OR TS=(carrelizumab)) OR TS=(SHR-1210)) OR TS=(SHR 1210)) OR TS=(GLS-010)) OR TS=(Cemiplimab)) OR TS=(REGN2810)) OR TS=(Sintilimab)) OR TS=(IBI308)) OR TS=(IBI-308)) OR TS=(Zimberelimab)) OR	68436 results

	TS=(Prolgolimab)) OR TS=(Dostarlimab)) OR TS=(Jemperli)) OR TS=(dostarlimab-gxly)) OR TS=(TSR-042)) OR TS=(GSK4057190)) OR TS=(PD-L1 inhibitor)) OR TS=(PD-L1 Inhibitors)) OR TS=(PD L1 Inhibitors)) OR TS=(PD-L1 Inhibitor)) OR TS=(PD L1 Inhibitor)) OR TS=(Programmed Death-Ligand 1 Inhibitors)) OR TS=(Programmed Death Ligand 1 Inhibitors)) OR TS=(PD-1-PD-L1 Blockade)) OR TS=(Blockade, PD-1-PD-L1)) OR TS=(PD 1 PD L1 Blockade)) OR TS=(Atezolizumab)) OR TS=(anti-PDL1)) OR TS=(immunoglobulin G1, anti-(human CD antigen CD274) (human monoclonal MDPL3280a heavy chain), disulfide with human monoclonal MDPL3280a kappa-chain, dimer)) OR TS=(MPDL3280A)) OR TS=(MPDL-3280A)) OR TS=(Tecentriq)) OR TS=(RG7446)) OR TS=(RG-7446)) OR TS=(Durvalumab)) OR TS=(MEDI4736)) OR TS=(MEDI-4736)) OR TS=(Imfinzi)) OR TS=(Avelumab)) OR TS=(MSB-0010682)) OR TS=(MSB0010682)) OR TS=(bavencio)) OR TS=(MSB0010718C)) OR TS=(MSB-0010718C)	
2	TS=(Rectal Neoplasm or Rectal Neoplasms or Rectum Neoplasm or Rectum Neoplasms or Neoplasm, Rectum or Neoplasm, Rectal or Neoplasms, Rectal or Neoplasms, Rectum or Neoplasm of Rectum or Neoplasm of the Rectum or Rectum Cancer or Rectum Cancers or Rectal Cancer or Rectal Cancers or Cancer, Rectal or Cancer, Rectum or Cancers, Rectal or Cancers, Rectum or Cancer of Rectum or Cancer of the Rectum or Rectal Tumors or Rectal Tumor or Rectum Tumor or Rectum Tumors or Tumor, Rectal or Tumors, Rectal or Tumor, Rectum or Tumors, Rectum or Tumor of Rectum or Tumor of the Rectum or Rectal Carcinoma or Rectal Carcinomas or Rectum Carcinoma or Rectum Carcinomas or Carcinoma, Rectal or Carcinomas, Recta or Carcinoma, Rectum or Carcinomas, Rectum or Carcinoma of Rectum or Carcinoma of the Rectum)	75864 results
3	(((((((TS=(randomized controlled trial)) OR TS=(controlled clinical trial)) OR TS=(Randomized)) OR TS=(placebo)) OR TS=(clinical trials as topic)) OR TS=(randomly)) OR TS=(Trial)) OR TS=(Prospective)) OR TS=(Retrospective)	3,913,138 results
4	TS=(Neoadjuvant)	70,991 results
5	1 AND 2 AND 3 AND 4	92 results

Embase		
No.	Query	Results
1	'pd-1 inhibitors':ab,ti OR 'pd 1 inhibitors':ab,ti OR 'pd-1 inhibitor':ab,ti OR 'inhibitor, pd-1':ab,ti OR 'pd 1 inhibitor':ab,ti OR 'programmed cell death protein 1 inhibitors':ab,ti OR 'programmed cell death protein 1 inhibitor':ab,ti OR pembrolizumab:ab,ti OR 'sch 900475':ab,ti OR lambrolizumab:ab,ti OR 'mk 3475':ab,ti OR keytruda:ab,ti OR nivolumab:ab,ti OR opdivo:ab,ti OR 'ono 4538':ab,ti OR 'mdx 1106':ab,ti OR mdx1106:ab,ti OR 'bms	50870 results

	936558':ab,ti OR bms936558:ab,ti OR toripalimab:ab,ti OR 'bgb a317':ab,ti OR camrelizumab:ab,ti OR carrelizumab:ab,ti OR 'shr 1210':ab,ti OR 'gls 010':ab,ti OR cemiplimab:ab,ti OR regn2810:ab,ti OR sintilimab:ab,ti OR ibi308:ab,ti OR 'ibi 308':ab,ti OR zimberelimab:ab,ti OR prolgolimab:ab,ti OR dostarlimab:ab,ti OR jemperli:ab,ti OR 'dostarlimab gxly':ab,ti OR 'tsr 042':ab,ti OR gsk4057190:ab,ti OR 'pd-l1 inhibitors':ab,ti OR 'pd l1 inhibitors':ab,ti OR 'pd-l1 inhibitor':ab,ti OR 'pd l1 inhibitor':ab,ti OR 'programmed death-ligand 1 inhibitors':ab,ti OR 'programmed death ligand 1 inhibitors':ab,ti OR 'pd-1-pd-l1 blockade':ab,ti OR 'blockade, pd-1-pd-l1':ab,ti OR 'pd 1 pd l1 blockade':ab,ti OR atezolizumab:ab,ti OR 'anti pdl1':ab,ti OR ('immunoglobulin g1, anti-':ab,ti AND 'human cd antigen cd274':ab,ti AND 'human monoclonal mdpl3280a heavy chain':ab,ti AND ', disulfide with human monoclonal mdpl3280a kappa-chain, dimer':ab,ti) OR mpdl3280a:ab,ti OR 'mpdl 3280a':ab,ti OR tecentriq:ab,ti OR rg7446:ab,ti OR 'rg 7446':ab,ti OR durvalumab:ab,ti OR medi4736:ab,ti OR 'medi 4736':ab,ti OR imfinzi:ab,ti OR avelumab:ab,ti OR 'msb 0010682':ab,ti OR msb0010682:ab,ti OR bavencio:ab,ti OR msb0010718c:ab,ti OR 'msb 0010718c':ab,ti	
2	'rectal neoplasm':ti,ab,kw OR 'rectal neoplasms':ti,ab,kw OR 'rectum neoplasm':ti,ab,kw OR 'rectum neoplasms':ti,ab,kw OR 'neoplasm, rectum':ti,ab,kw OR 'neoplasm, rectal':ti,ab,kw OR 'neoplasms, rectal':ti,ab,kw OR 'neoplasms, rectum':ti,ab,kw OR 'neoplasm of rectum':ti,ab,kw OR 'neoplasm of the rectum':ti,ab,kw OR 'rectum cancer':ti,ab,kw OR 'rectum cancers':ti,ab,kw OR 'rectal cancer':ti,ab,kw OR 'rectal cancers':ti,ab,kw OR 'cancer, rectal':ti,ab,kw OR 'cancer, rectum':ti,ab,kw OR 'cancers, rectal':ti,ab,kw OR 'cancers, rectum':ti,ab,kw OR 'cancer of rectum':ti,ab,kw OR 'cancer of the rectum':ti,ab,kw OR 'rectal tumors':ti,ab,kw OR 'rectal tumor':ti,ab,kw OR 'rectum tumor':ti,ab,kw OR 'rectum tumors':ti,ab,kw OR 'tumor, rectal':ti,ab,kw OR 'tumors, rectal':ti,ab,kw OR 'tumor, rectum':ti,ab,kw OR 'tumors, rectum':ti,ab,kw OR 'tumor of rectum':ti,ab,kw OR 'tumor of the rectum':ti,ab,kw OR 'rectal carcinoma':ti,ab,kw OR 'rectal carcinomas':ti,ab,kw OR 'rectum carcinoma':ti,ab,kw OR 'rectum carcinomas':ti,ab,kw OR 'carcinoma, rectal':ti,ab,kw OR 'carcinomas, recta':ti,ab,kw OR 'carcinoma, rectum':ti,ab,kw OR 'carcinomas, rectum':ti,ab,kw OR 'carcinoma of rectum':ti,ab,kw OR 'carcinoma of the rectum':ti,ab,kw	16466results
3	'randomized controlled trial':ab,ti OR 'controlled clinical trial':ab,ti OR randomized:ab,ti OR placebo:ab,ti OR 'clinical trials as topic':ab,ti OR randomly:ab,ti OR trial:ab,ti OR prospective:ab,ti OR retrospective:ab,ti	4208427results
4	neoadjuvant:ab,ti	94,375results
5	1 AND 2 AND 3 AND 4	104 results

cochrane library		
No.	Query	Results
#1	MeSH descriptor: [Rectal Neoplasms] explode all trees	2510results
#2	(Rectal Neoplasm or Rectal Neoplasms or Rectum Neoplasm or Rectum Neoplasms or Neoplasm, Rectum or Neoplasm, Rectal or Neoplasms, Rectal or Neoplasms, Rectum or Neoplasm of Rectum or Neoplasm of the Rectum or Rectum Cancer or Rectum Cancers or Rectal Cancer or Rectal Cancers or Cancer, Rectal or Cancer, Rectum or Cancers, Rectal or Cancers, Rectum or Cancer of Rectum or Cancer of the Rectum or Rectal Tumors or Rectal Tumor or Rectum Tumor or Rectum Tumors or Tumor, Rectal or Tumors, Rectal or Tumor, Rectum or Tumors, Rectum or Tumor of Rectum or Tumor of the Rectum or Rectal Carcinoma or Rectal Carcinomas or Rectum Carcinoma or Rectum Carcinomas or Carcinoma, Rectal or Carcinomas, Recta or Carcinoma, Rectum or Carcinomas, Rectum or Carcinoma of Rectum or Carcinoma of the Rectum):ti,ab,kw	9106 results
#3	#1 or #2	9246 results
#4	MeSH descriptor: [Immune Checkpoint Inhibitors] explode all trees	205 results
#5	(PD-1Inhibitors OR PD-1 Inhibitors OR PD 1 Inhibitors OR PD-1 Inhibitor OR Inhibitor, PD-1 OR PD 1 Inhibitor OR Programmed Cell Death Protein 1 Inhibitor OR Programmed Cell Death Protein 1 Inhibitors OR Nivolumab OR Opdivo OR ONO-4538 OR ONO 4538 OR ONO4538 OR MDX-1106 OR MDX 1106 OR MDX1106 OR BMS-936558 OR BMS 936558 OR BMS936558 OR Pembrolizumab OR SCH-900475 OR lambrolizumab OR MK-3475 OR Keytruda OR Tislelizumab OR BGB-A317 OR Toripalimab OR Camrelizumab OR carrelizumab OR SHR-1210 OR SHR 1210 OR Sintilimab OR IBI 308 OR IBI308 OR IBI-308 ORZimberelimab OR GLS-010 ORProlgolimab OR Dostarlimab OR Jemperli OR dostarlimab-gxly OR TSR-042 OR GSK4057190 OR PD-L1 Inhibitors OR PD-L1 Inhibitors OR PD L1 Inhibitors OR PD-L1 Inhibitor OR PD L1 Inhibitor OR Programmed Death-Ligand 1 Inhibitors OR Programmed Death Ligand 1 Inhibitors OR PD-1 PD-L1 Blockade OR Blockade, PD-1 PD-L1 OR PD 1 PD L1 Blockade OR Atezolizumab OR anti-PDL1 OR immunoglobulin G1, anti-(human CD antigen CD274) (human monoclonal MDPL3280a heavy chain), disulfide with human monoclonal MDPL3280a kappa-chain, dimer OR MPDL3280A OR MPDL-3280A OR Tecentriq OR RG7446 OR RG-7446 OR Durvalumab OR MEDI4736 OR MEDI-4736 OR Imfinzi OR Avelumab OR MSB-0010682 OR MSB0010718C OR	13954 results

	MSB-0010718C OR MSB0010682 OR bavencio):ti,ab,kw	
#6	#4 or #5	13992 results
#7	(Neoadjuvant):ti,ab,kw	12048 results
#8	(randomized controlled trial OR controlled clinical trial OR Randomized OR placebo OR clinical trials as topic OR randomly OR Trial OR Prospective OR Retrospective):ti,ab,kw	1513288 results
#9	#3 and #6 and #7 and #8	58 results
