# Supplementary material for: Efficacy and safety of neoadjuvant PD-1 inhibitors or PD-L1 inhibitors combined with chemoradiotherapy for locally advanced rectal cancer: a systematic review and meta-analysis
Source: Front Pharmacol. 2025 May 16;16:1570467. doi: 10.3389/fphar.2025.1570467 (PMC12122451; doi:10.3389/fphar.2025.1570467)
Supplement: Supplementary file 4 [file Image1.pdf]

Fig S1. Sensitivity analysis of the meta-analysis on pCR.

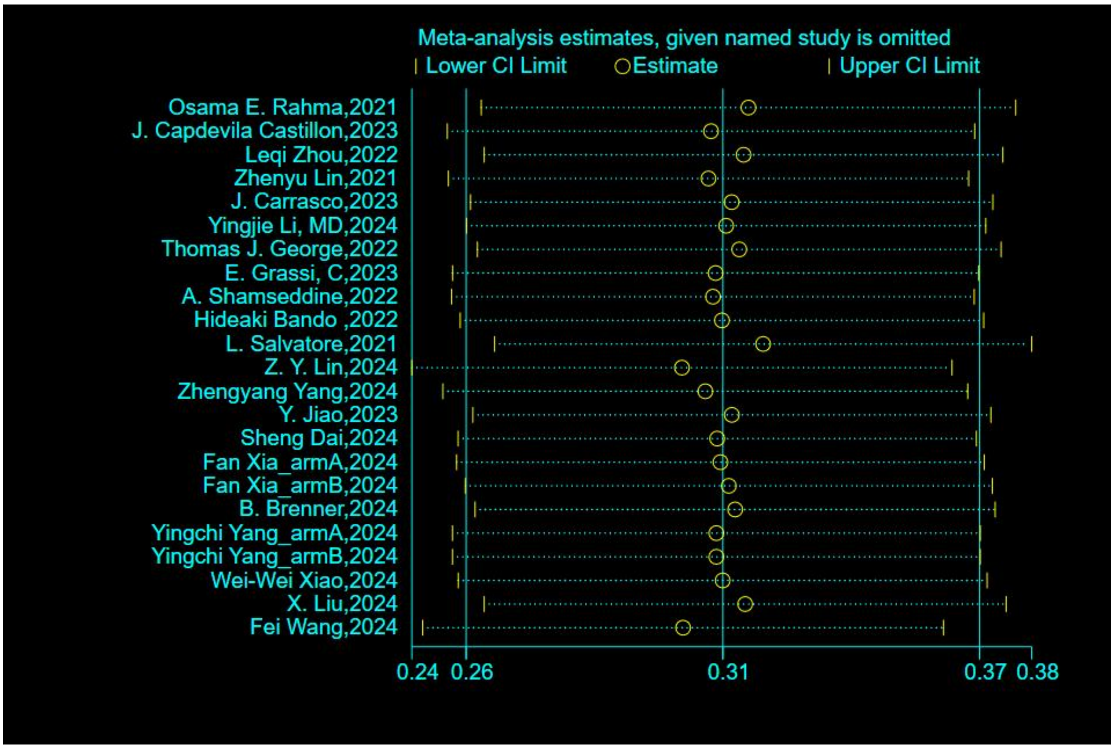

Fig S2. Funnel plot assessing publication bias in the meta-analysis of pCR.

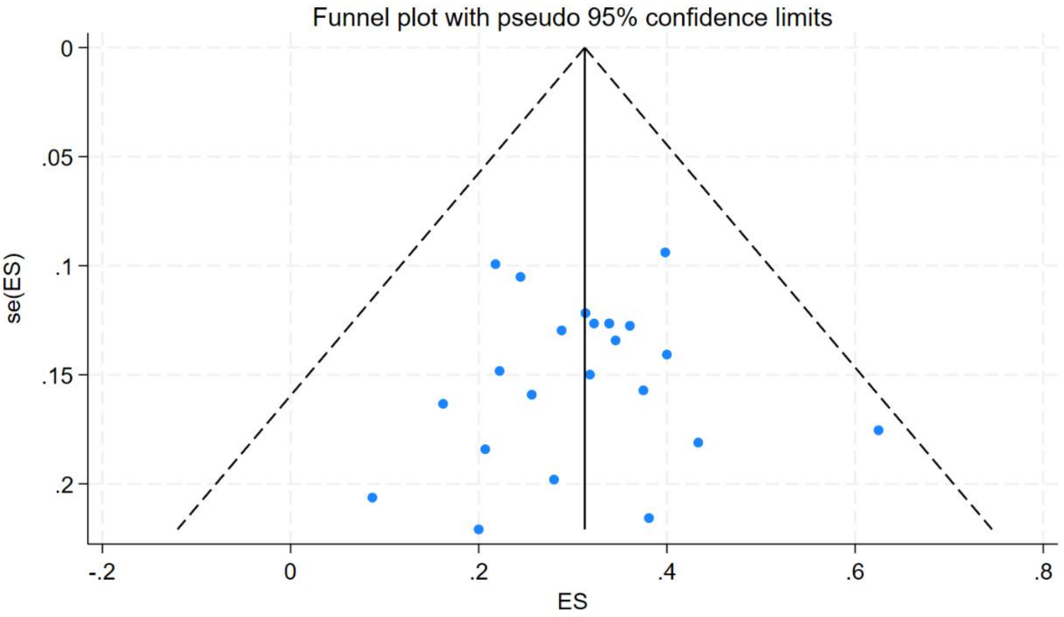

Fig S3. Sensitivity analysis of the meta-analysis on cCR.

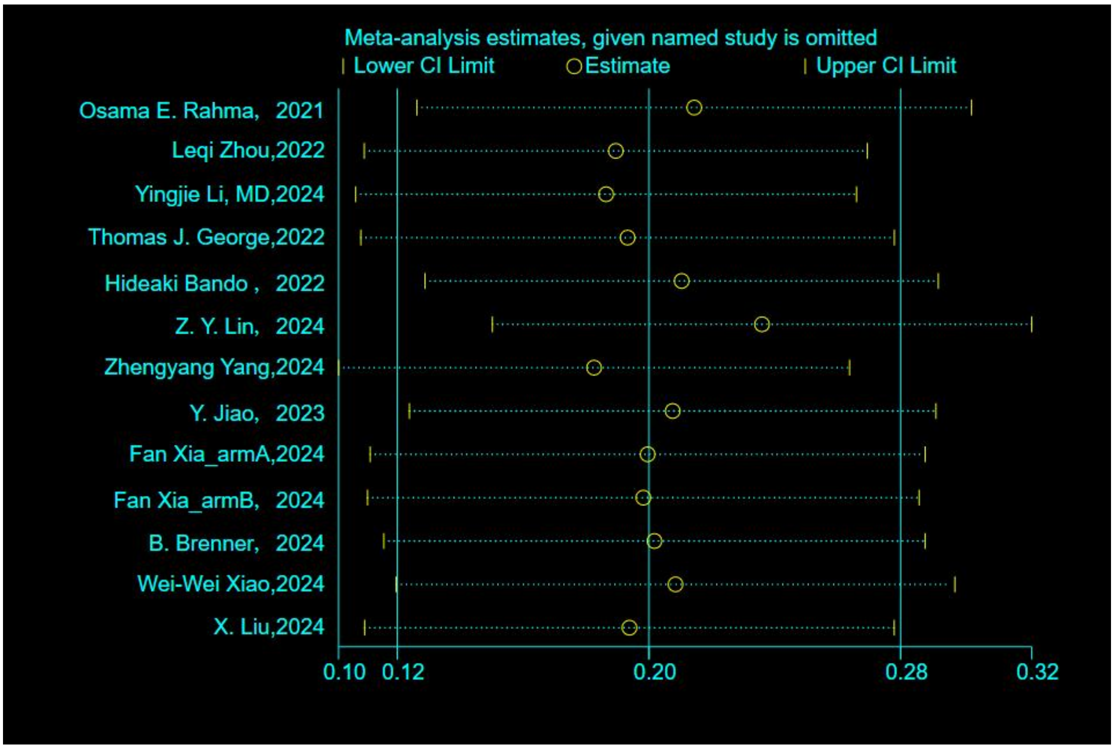

Fig S4. Funnel plot assessing publication bias in the meta-analysis of cCR.

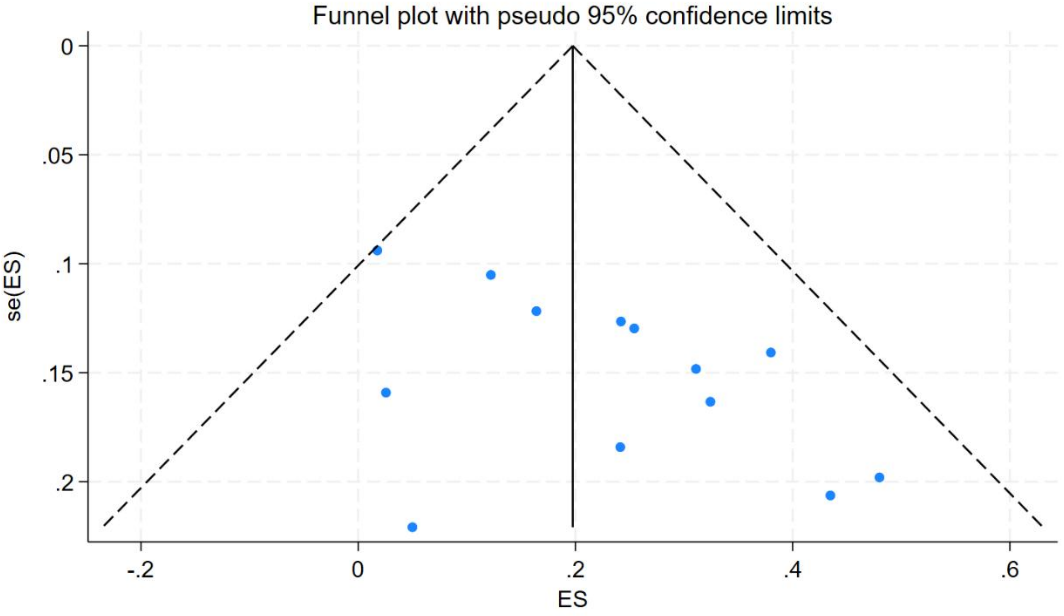

Fig S5. Sensitivity analysis of the meta-analysis on MPR.

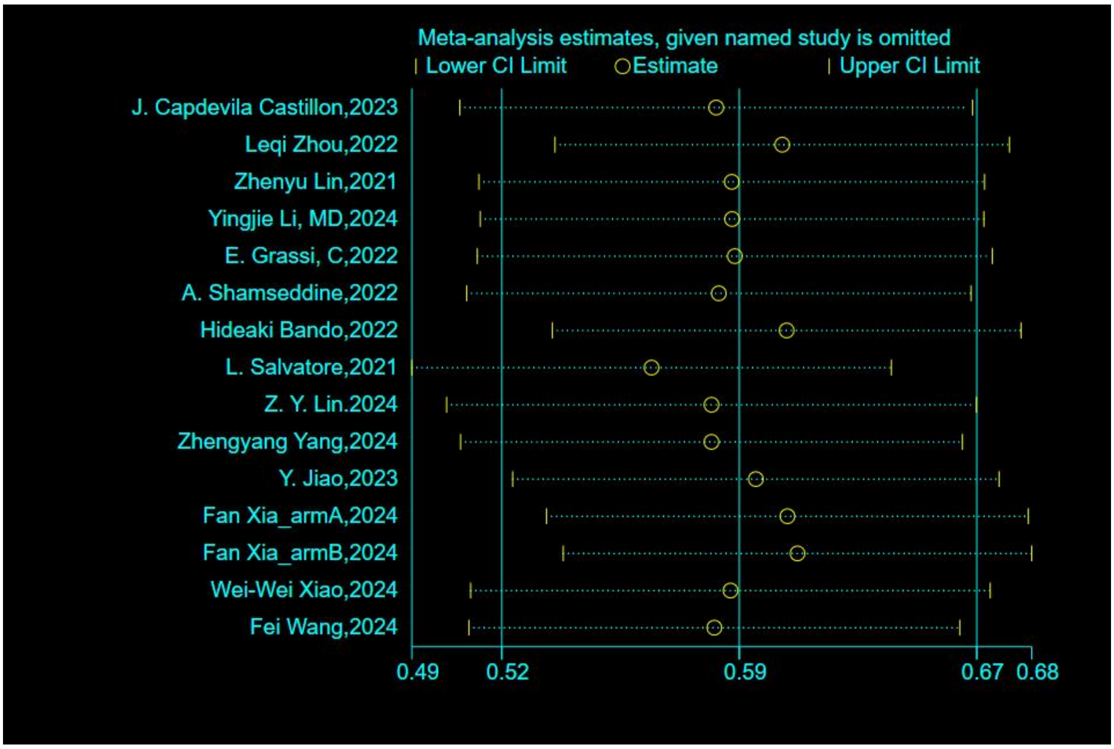

Fig S6. Funnel plot assessing publication bias in the meta-analysis of MPR.

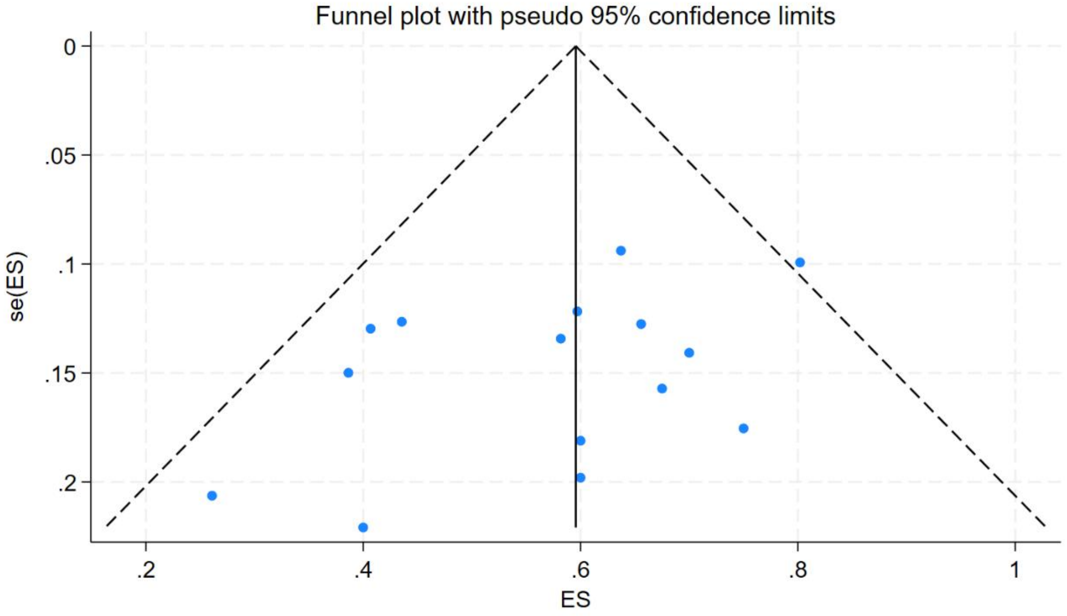

Fig S7. Sensitivity analysis of the meta-analysis on surgery rate.

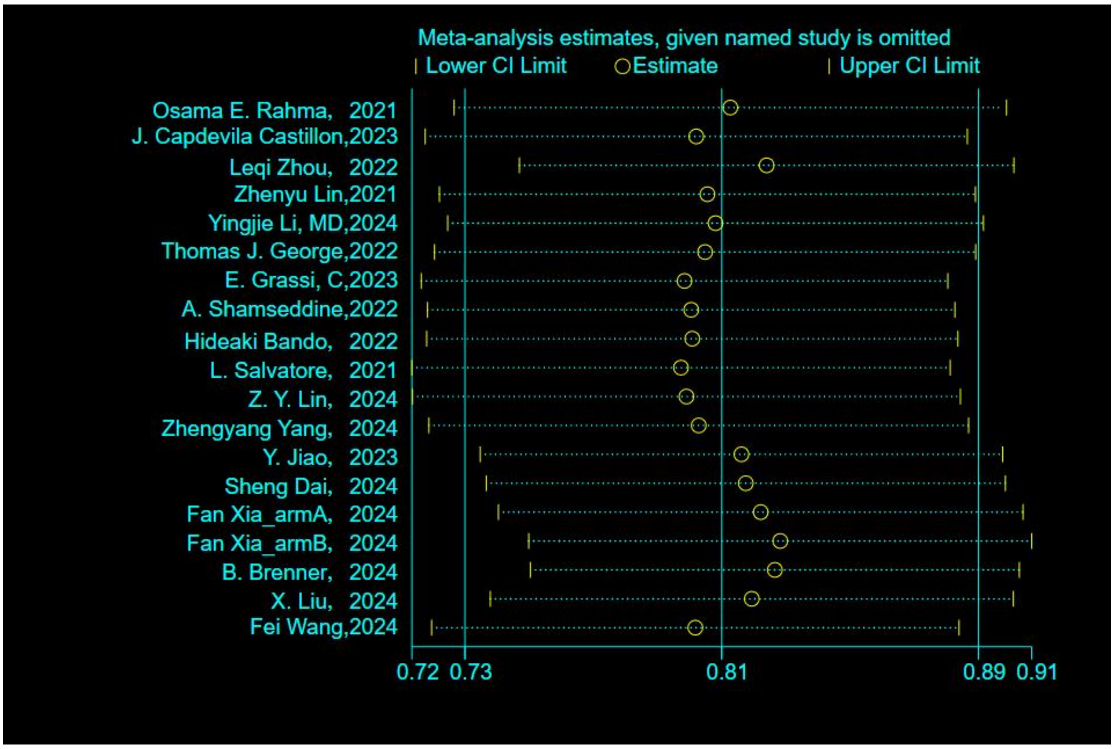

Fig S8. Funnel plot assessing publication bias in the meta-analysis of surgery rate.

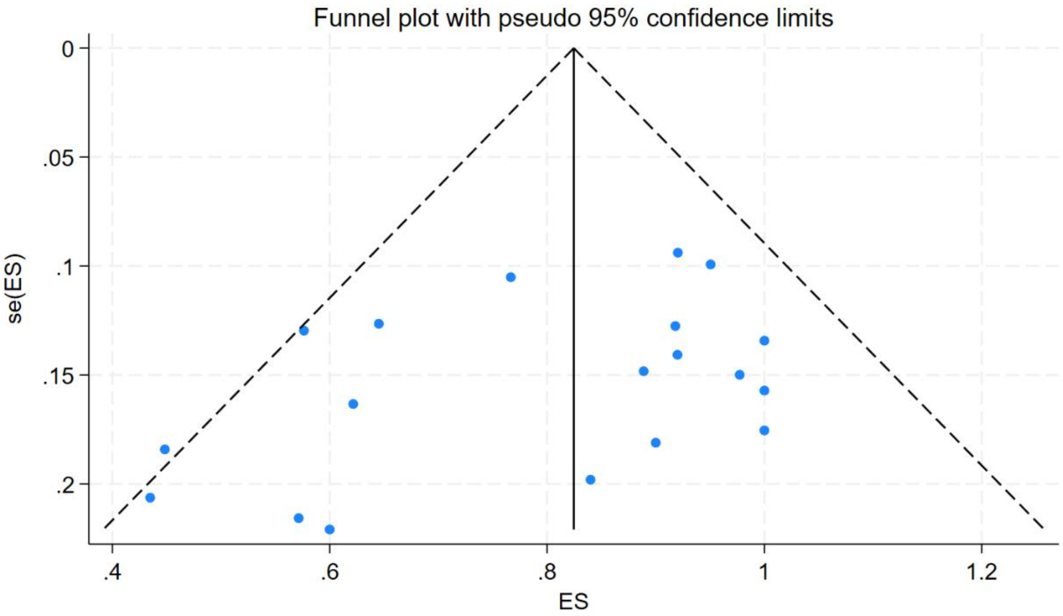

Fig S9. Sensitivity analysis of the meta-analysis on R0 resection rate.

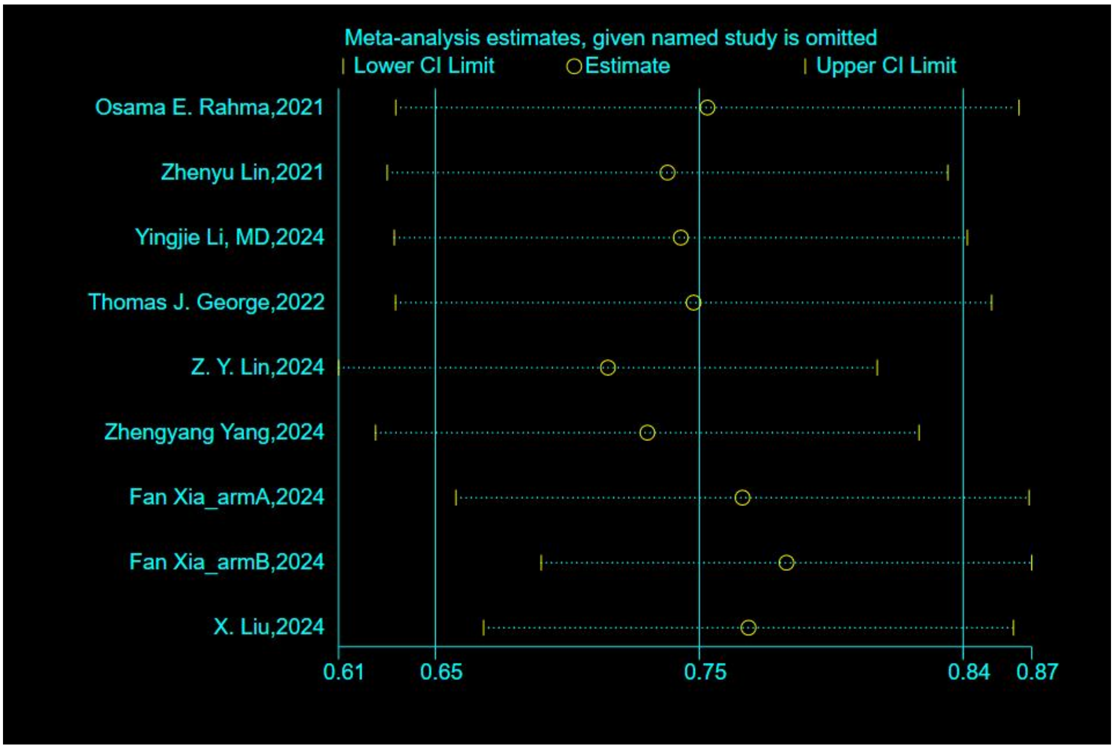

Fig S10. Funnel plot assessing publication bias in the meta-analysis of R0 resection rate.

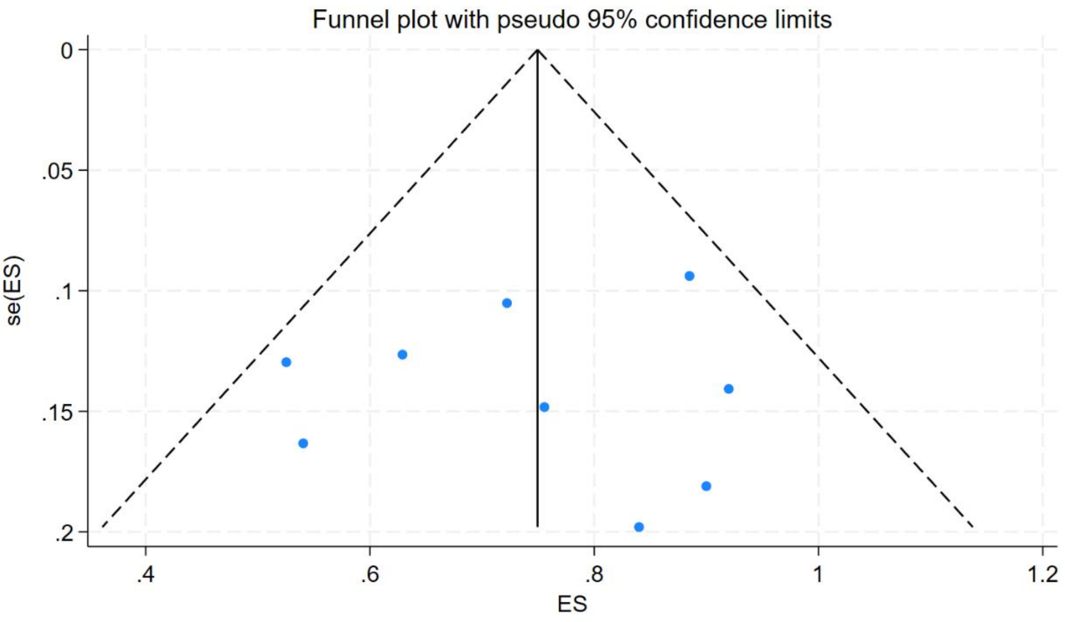

Fig S11. Sensitivity analysis of the meta-analysis on SSS.

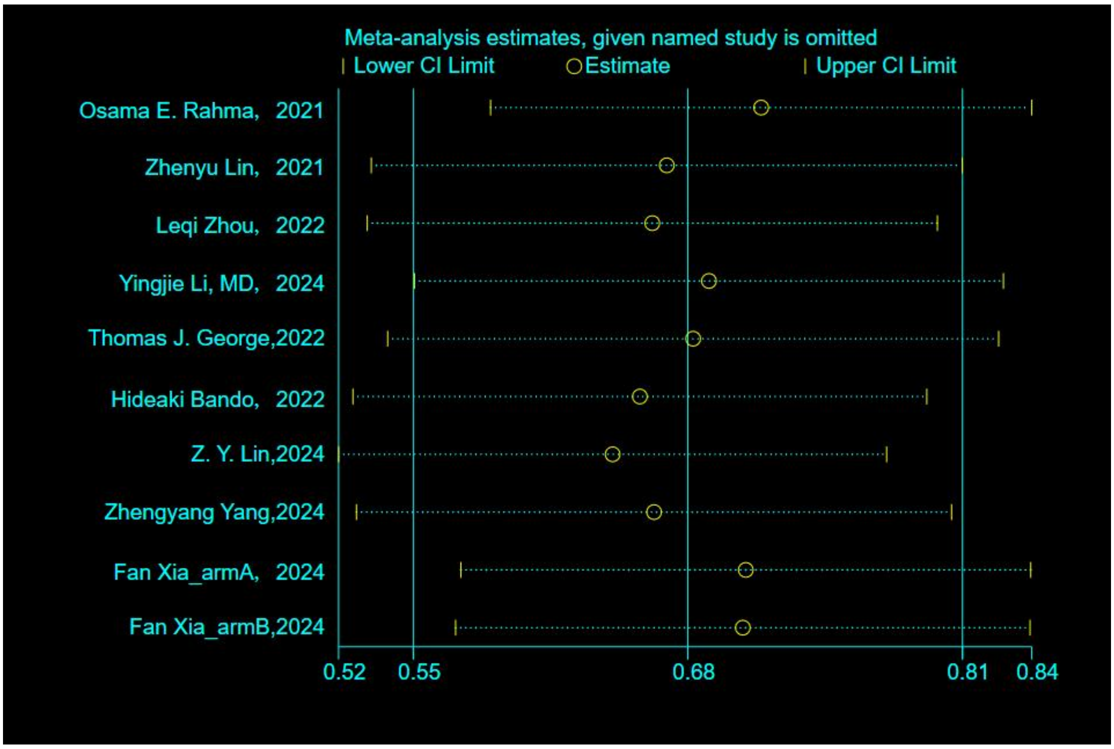

Fig S12. Funnel plot assessing publication bias in the meta-analysis of SSS.

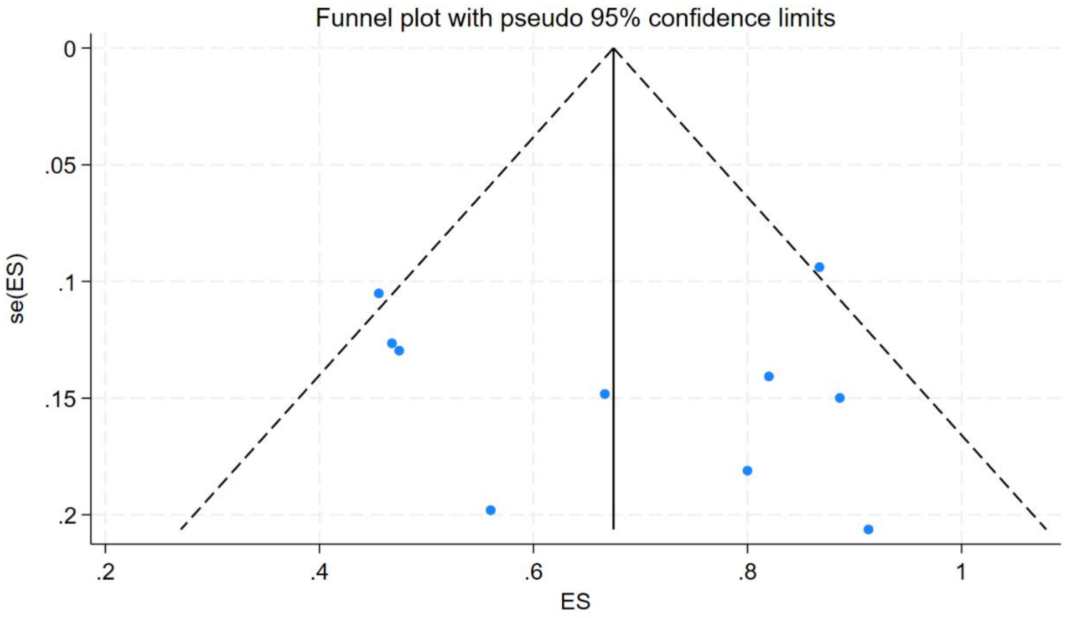

Fig S13. Sensitivity analysis of the meta-analysis on 3 years DFS.

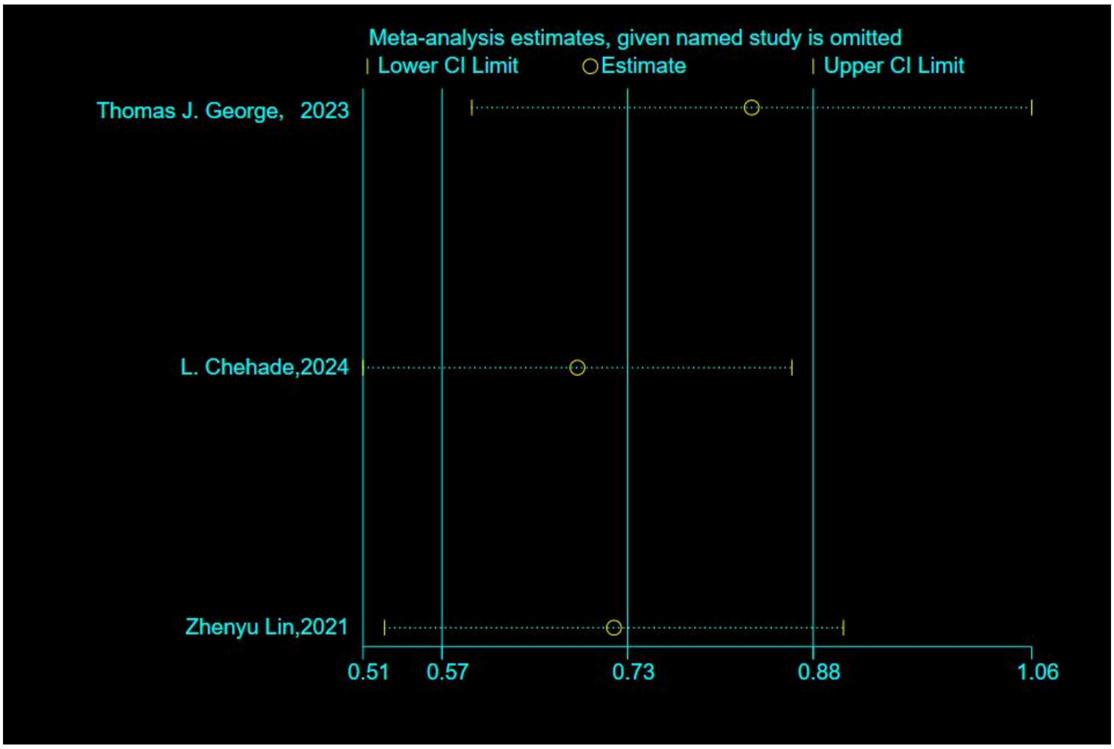

Fig S14. Funnel plot assessing publication bias in the meta-analysis of 3 years DFS.

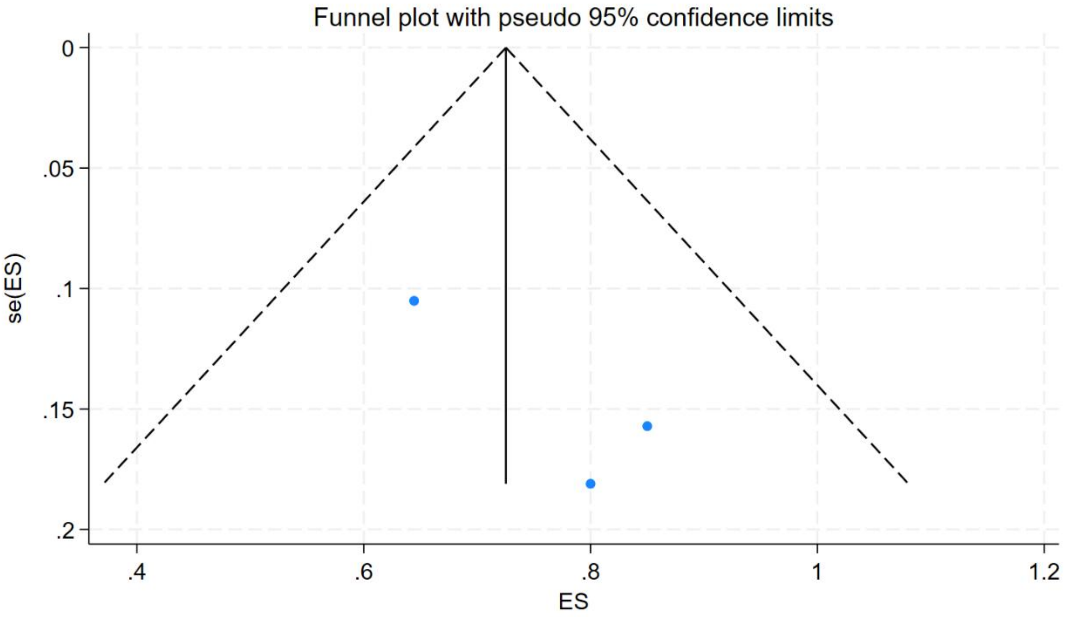

Fig S15. Sensitivity analysis of the meta-analysis on Grade ≥ 3 AEs.

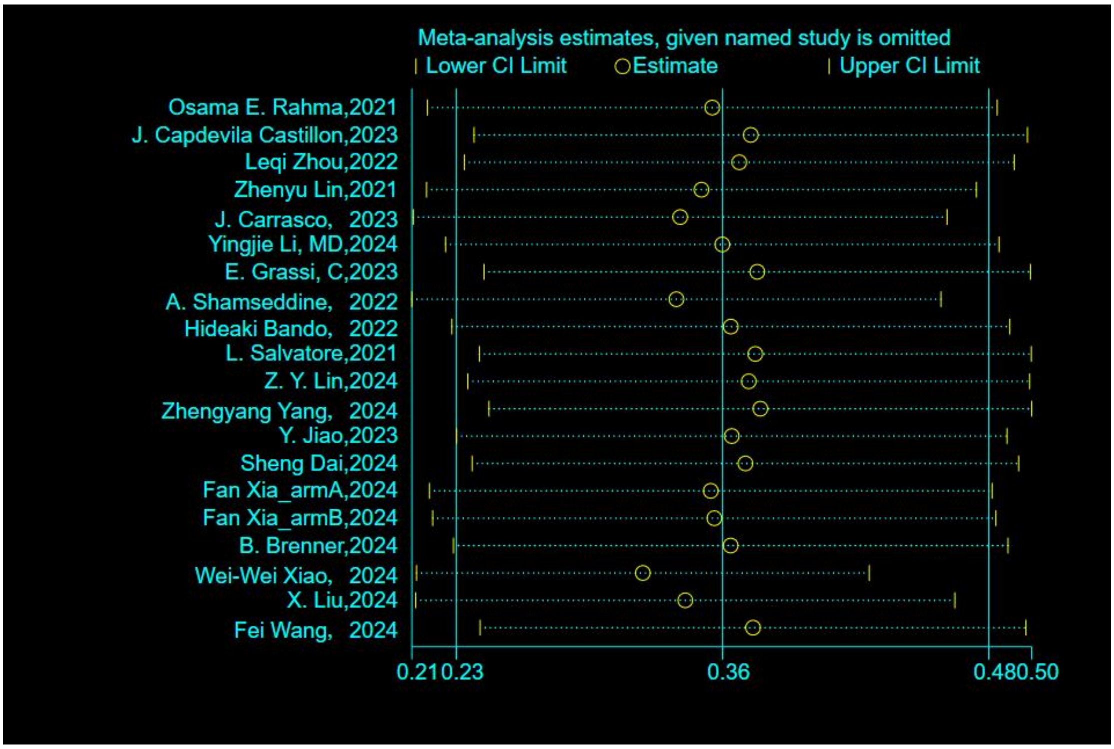

Fig S16. Funnel plot assessing publication bias in the meta-analysis of Grade ≥ 3 AEs.

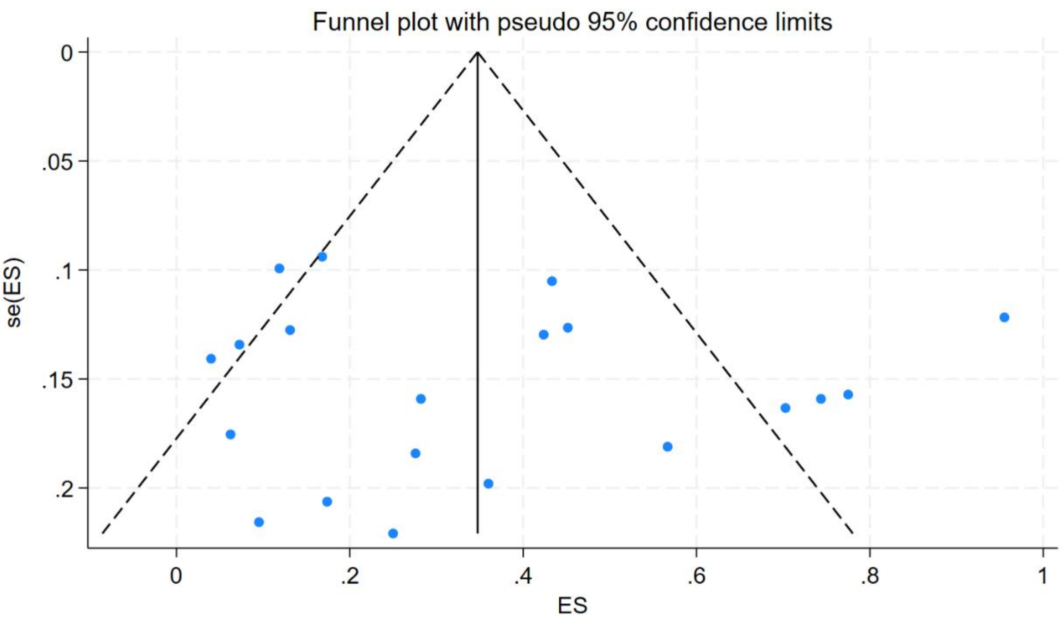

Fig S17. Forest plot of the meta-analysis of MMR status based on pCR.

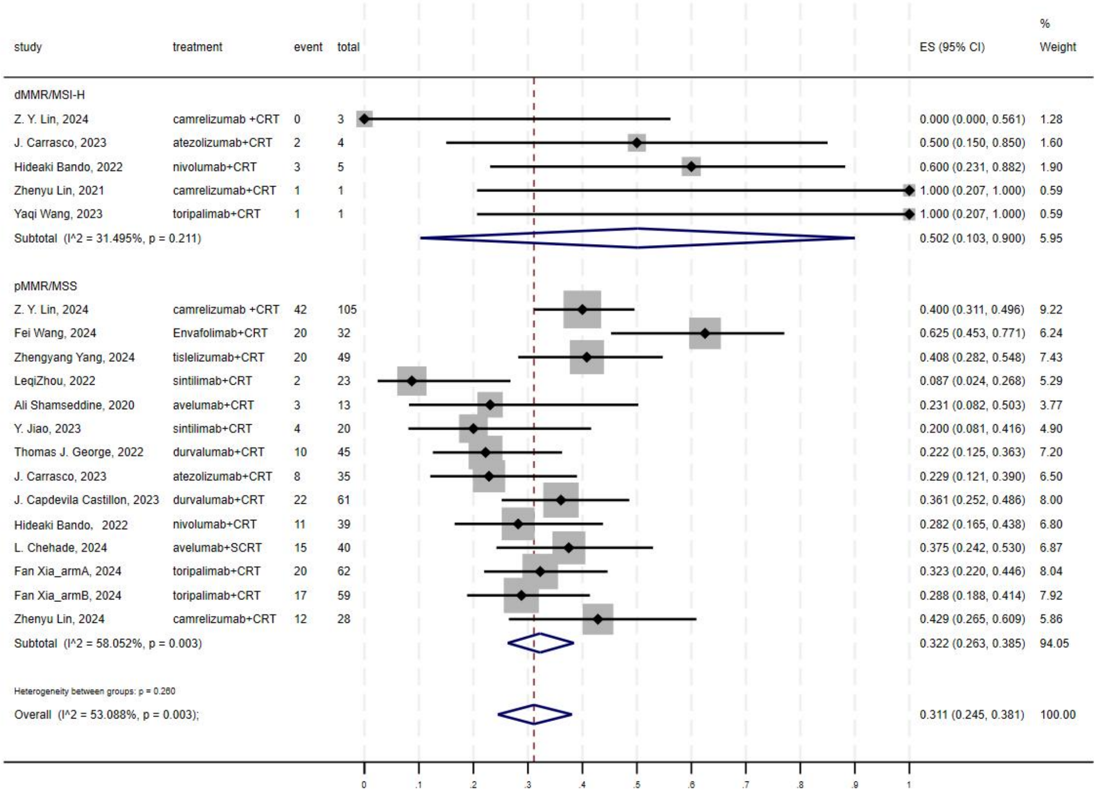

Fig S18. Forest plot of the meta-analysis of MMR status based on MPR.

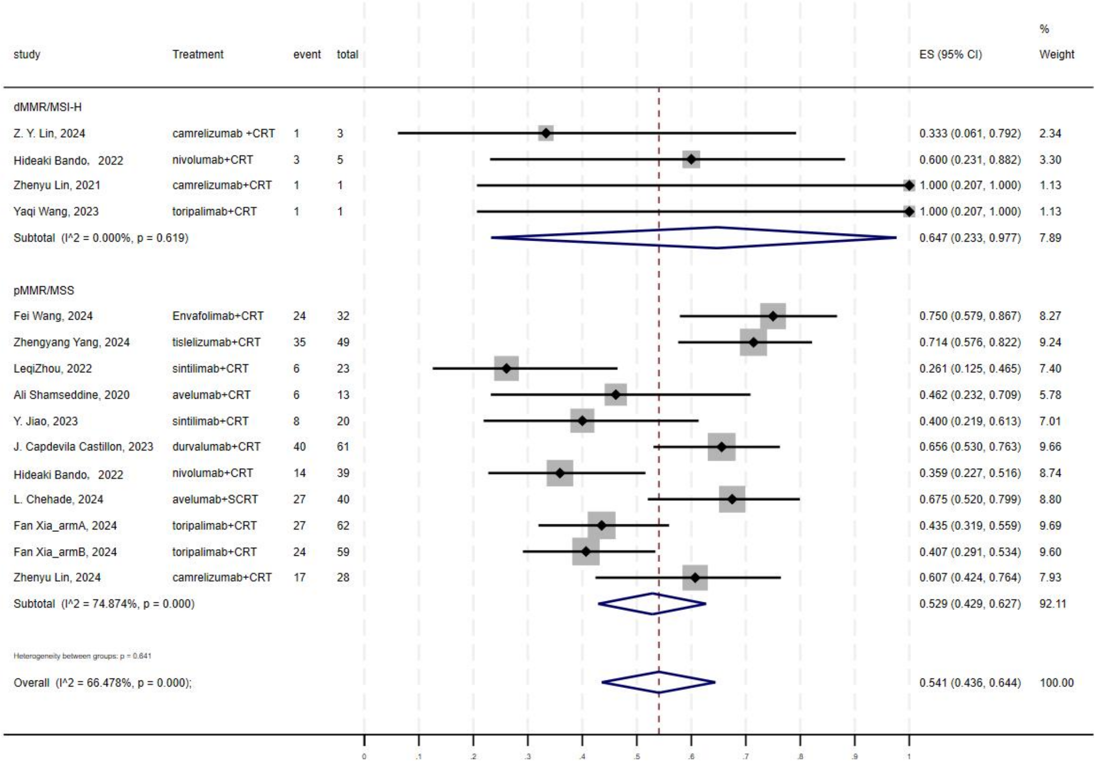

Fig S19. Meta-regression of pCR rates by MMR status.

```
Meta-regression                                    Number of obs  =       19
REML estimate of between-study variance            tau2           =        0
% residual variation due to heterogeneity          I-squared_res  =    0.00%
Proportion of between-study variance explained     Adj R-squared  =       .%
With Knapp-Hartung modification

------------------------------------------------------------------------------
         _ES | Coefficient  Std. err.      t    P>|t|     [95% conf. interval]
-------------+----------------------------------------------------------------
       group |  -.1812411   .2494478    -0.73   0.477    -.7075299    .3450476
       _cons |    .699423   .4940064     1.42   0.175    -.3428395    1.741685
------------------------------------------------------------------------------
```

Fig S20. Meta-regression of MPR rates by MMR status.

```
Meta-regression                                    Number of obs  =       15
REML estimate of between-study variance            tau2           =  .001202
% residual variation due to heterogeneity          I-squared_res  =    0.00%
Proportion of between-study variance explained     Adj R-squared  =  -34.62%
With Knapp-Hartung modification

------------------------------------------------------------------------------
         _ES | Coefficient  Std. err.      t    P>|t|     [95% conf. interval]
-------------+----------------------------------------------------------------
       group |  -.0876563   .2935484    -0.30   0.770    -.7218291    .5465166
       _cons |   .7101296   .5808157     1.22   0.243    -.5446465    1.964906
------------------------------------------------------------------------------

.
```

Fig S21. Forest plot of the meta-analysis of radiotherapy strategies based on pCR.

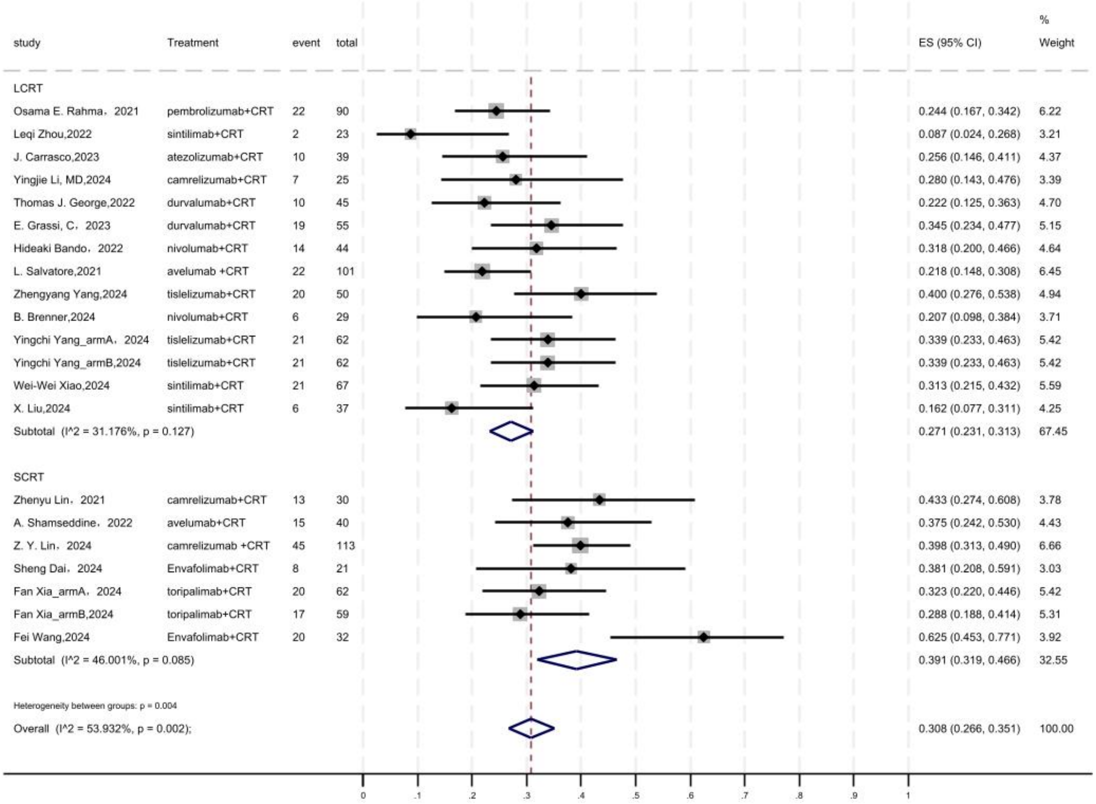

Fig S22. Meta-regression of pCR rates by radiotherapy strategies.

Meta-regression		Number of obs =	21
**REML** estimate of between-study variance		tau2 =	**0**
% residual variation due to heterogeneity		I-squared_res =	**0.00%**
Proportion of between-study variance explained		Adj R-squared =	.%
**With** Knapp-Hartung modification			

_ES	Coefficient	Std. err.	t	P>|t|	[95% conf.	interval]
group	**.1110846**	**.0642855**	**1.73**	**0.100**	**-.0234664**	**.2456356**
_cons	**.1645481**	**.0906019**	**1.82**	**0.085**	**-.0250839**	**.3541801**

.

Fig S23. Forest plot of the meta-analysis of neoadjuvant therapy models based on pCR.

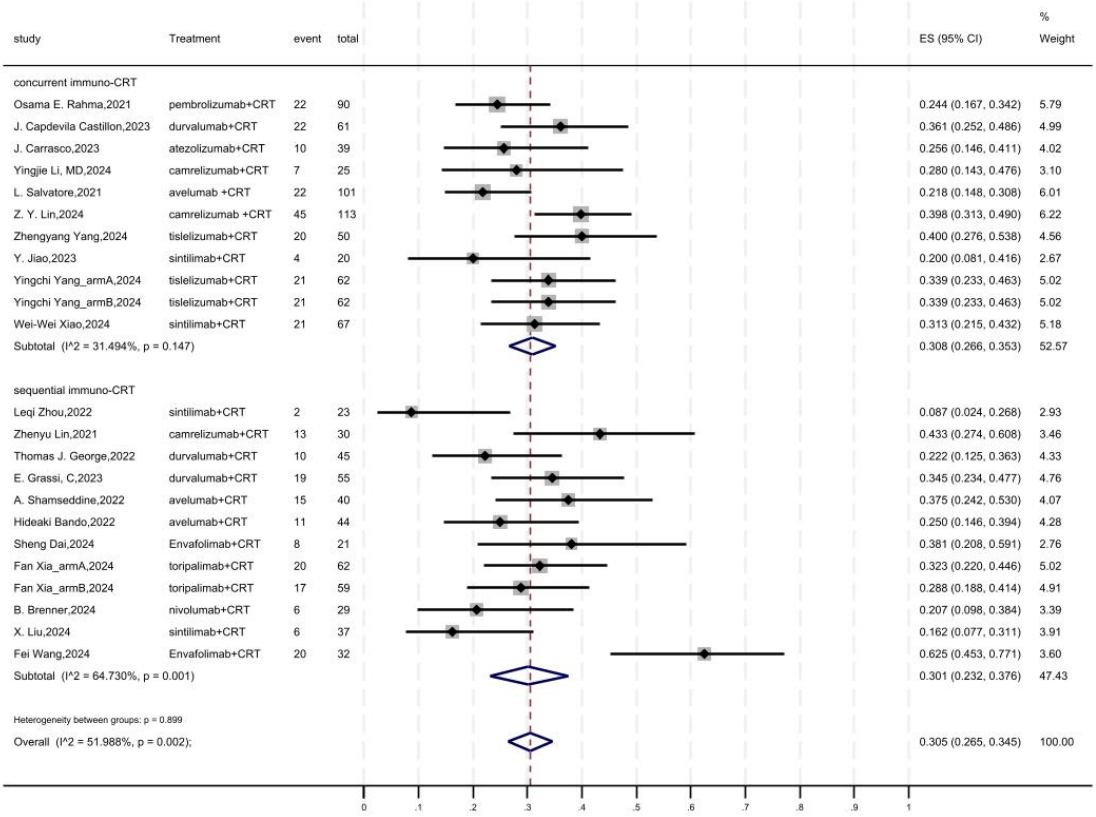

Fig S24. Meta-regression of pCR rates by neoadjuvant therapy models.

```
Meta-regression                                      Number of obs   =       23
REML estimate of between-study variance              tau2            =        0
% residual variation due to heterogeneity            I-squared_res   =    0.00%
Proportion of between-study variance explained       Adj R-squared   =       .%
With Knapp-Hartung modification
```

_ES	Coefficient	Std. err.	t	P>|t|	[95% conf. interval]	
group	-.0033606	.0592301	-0.06	0.955	-.1265364	.1198152
_cons	.3148979	.0884401	3.56	0.002	.1309767	.4988192

.

Fig S25. Forest plot of the meta-analysis of PD-1/PD-L1 inhibitors based on pCR.

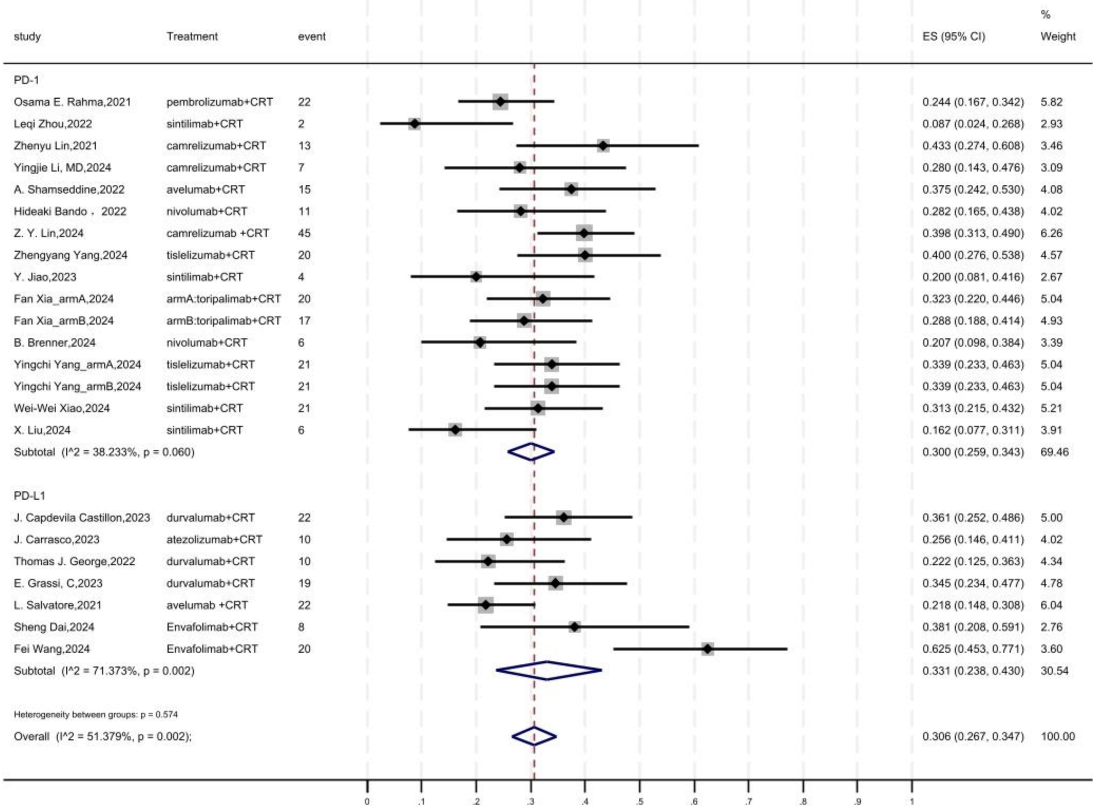

Fig S26. Forest plot of the meta-analysis of PD-1/PD-L1 inhibitors based on Grade ≥3 AEs.

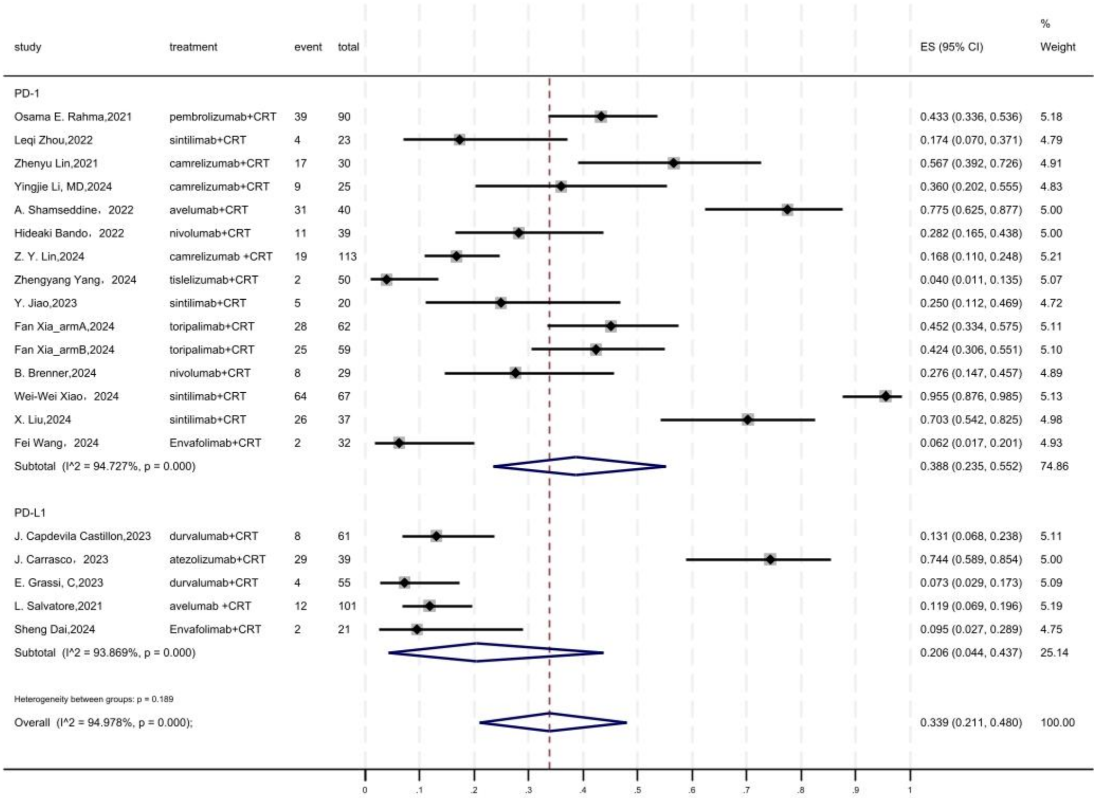

Fig S27. Meta-regression of pCR rates by PD-1/PD-L1 inhibitors.

```
Meta-regression                                   Number of obs  =       23
REML estimate of between-study variance           tau2           =        0
% residual variation due to heterogeneity         I-squared_res  =    0.00%
Proportion of between-study variance explained    Adj R-squared  =       .%
With Knapp-Hartung modification
```

_ES	Coefficient	Std. err.	t	P>|t|	[95% conf. interval]	
group	.0033981	.0634247	0.05	0.958	-.1285008	.135297
_cons	.3070618	.0877449	3.50	0.002	.1245864	.4895373

.

Fig S28. Meta-regression of Grade ≥3 AEs by PD-1/PD-L1 inhibitors.

```
Meta-regression                                      Number of obs   =        20
REML estimate of between-study variance              tau2            =     .0522
% residual variation due to heterogeneity            I-squared_res   =    71.83%
Proportion of between-study variance explained       Adj R-squared   =     5.49%
With Knapp-Hartung modification
```

_ES	Coefficient	Std. err.	t	P>|t|	[95% conf. interval]	
group	-.1754581	.1407874	-1.25	0.229	-.4712414	.1203252
_cons	.5777515	.1875123	3.08	0.006	.1838028	.9717002

Fig S29. Forest plot of the meta-analysis of type of trials based on pCR.

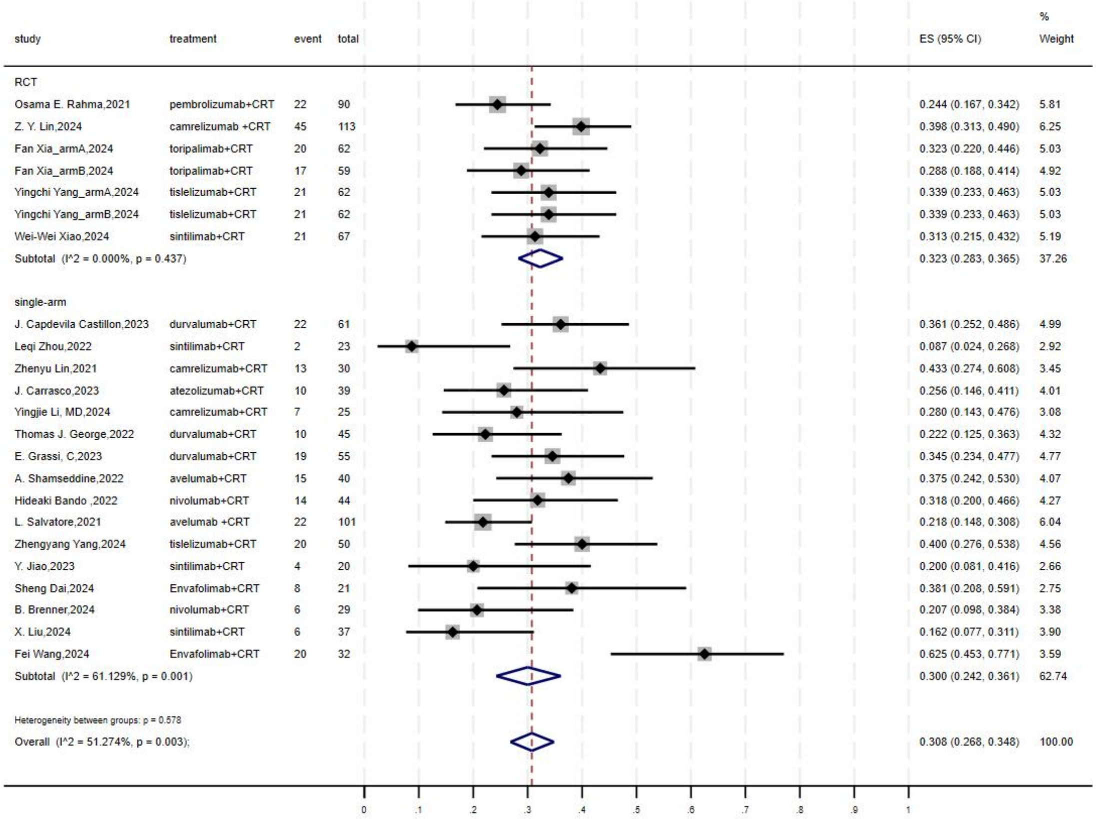

Fig S30. Meta-regression of pCR rates by type of trials.

```
Meta-regression                                    Number of obs   =       23
REML estimate of between-study variance            tau2            =        0
% residual variation due to heterogeneity          I-squared_res   =    0.00%
Proportion of between-study variance explained     Adj R-squared   =       .%
With Knapp-Hartung modification
```

_ES	Coefficient	Std. err.	t	P>|t|	[95% conf. interval]	
group	-.0205569	.0586838	-0.35	0.730	-.1425966	.1014828
_cons	.344804	.0960714	3.59	0.002	.1450126	.5445954

.
